# USP22 upregulates ZEB1-mediated VEGFA transcription in hepatocellular carcinoma

**DOI:** 10.1038/s41419-023-05699-y

**Published:** 2023-03-11

**Authors:** Kai Zeng, Weiwen Xie, Chunyu Wang, Shengli Wang, Wei Liu, Yingjie Su, Lin Lin, Renlong Zou, Ge Sun, Baosheng Zhou, Manlin Wang, Ruina Luan, Yu Bai, Yunlong Huo, Shigeaki Kato, Xinping Zhong, Yue Zhao

**Affiliations:** 1 Department of Cell Biology, Key laboratory of Cell Biology, Ministry of Public Health, and Key laboratory of Medical Cell Biology, Ministry of Education, School of Life Sciences, China Medical University, Shenyang, Liaoning Province PR China; 2 Department of Pathology, Shengjing Hospital of China Medical University, Shenyang, Liaoning Province PR China; 3 Graduate School of Life Science and Engineering, Iryo Sosei University, Iino, Chuo-dai, Iwaki, Fukushima, 9708551 Japan; 4 Research Institute of Innovative Medicine, Tokiwa Foundation, Iwaki, Fukushima, Japan; 5 Department of General Surgery, The First Affiliated Hospital of China Medical University, Shenyang, Liaoning Province PR China

**Keywords:** Oncogenes, Ubiquitylation, Transcriptional regulatory elements

## Abstract

Hepatocellular carcinoma (HCC) is a common solid tumor with high rate of recurrence and mortality. Anti-angiogenesis drugs have been used for the therapy of HCC. However, anti-angiogenic drug resistance commonly occurs during HCC treatment. Thus, identification of a novel VEGFA regulator would be better understanding for HCC progression and anti-angiogenic therapy resistance. Ubiquitin specific protease 22 (USP22) as a deubiquitinating enzyme, participates in a variety of biological processes in numerous tumors. While the molecular mechanism underlying the effects of USP22 on angiogenesis is still needed to be clarified. Here, our results demonstrated that USP22 acts as a co-activator of *VEGFA* transcription. Importantly, USP22 is involved in maintenance of ZEB1 stability via its deubiquitinase activity. USP22 was recruited to ZEB1-binding elements on the promoter of *VEGFA*, thereby altering histone H2Bub levels, to enhance ZEB1-mediated *VEGFA* transcription. USP22 depletion decreased cell proliferation, migration, Vascular Mimicry (VM) formation, and angiogenesis. Furthermore, we provided the evidence to show that knockdown of USP22 inhibited HCC growth in tumor-bearing nude mice. In addition, the expression of USP22 is positively correlated with that of ZEB1 in clinical HCC samples. Our findings suggest that USP22 participates in the promotion of HCC progression, if not all, at least partially via up-regulation of *VEGFA* transcription, providing a novel therapeutic target for anti-angiogenic drug resistance in HCC.

## Introduction

Hepatocellular carcinoma (HCC) is the main form of primary liver cancer, accounting for more than 80% of the total number [1]. The 5-year relative survival rate of HCC is only 20%, ranking the second in the world in terms of mortality [2]. According to statistics, more than 1 million patients will die of this disease by 2030 [3]. The number of liver cancer patients in our country ranks first in the world. Currently, there is few effective cures for HCC, and the recurrence rate is as high as 70% within 5 years after surgical resection [4]. Liver is the only organ in the body with dual arterial and venous blood supply, which makes HCC rely on adequate blood infusion for rapid proliferation or metastasis. Although currently anti-vascular drugs often occur therapeutic resistance [5] and could not completely cure HCC, anti-VEGF signaling pathway drugs such as sorafenib are still used as first-line therapy for HCC [1]. Therefore, anti-angiogenesis is one of the necessary strategies for the treatment of HCC, and looking for a novel VEGFA regulator that controls the expression of endogenous VEGFA involved in various tumor progression in HCC, would contribute to find a novel therapeutic target for HCC.

USP22 (Ubiquitin-specific Protease 22) is a member of the USP (Ubiquitin-specific Proteases) deubiquitination enzyme family [6]. USP22 as a core component of the SAGA (SPT-Ada-GCN5-acetyltransferase) complex mediates histone H2A/H2B deubiquitination to co-activate nuclear receptor-induced transcription [7, 8]. USP22 also removes histone H2A monoubiquitination to promote the transcription of genes required for invariant natural killer T (iNKT) cells development [9]. On the other hand, USP22 has also been considered to participate in deubiquitination of several non-histone substrates such as, PPARγ and PU.1 [10, 11]. USP22 stabilizes Cyclin D1 and Cyclin B1 to promote cell cycle progression [12]. USP22 deubiquitinates CD274 to suppress anticancer immunity [13]. USP22 maintains XPC (nucleotide excision repair protein) stability through deubiquitylation to promote cancer cells survival to DNA damage [14]. Our previously study has demonstrated that USP22 maintains estrogen receptor α (ERα) stability through its deubiquitination activity to co-activate ERα-mediated transactivation in breast cancer cells [15]. It has been reported that USP22 plays an important role in tumor drug resistance. USP22 can up-regulate the downstream stemness genes induced by HIF-1α under hypoxic conditions [16], and participate in the chemoresistance of HCC. USP22 up-regulates the SIRT1/AKT/MRP1 signaling pathway and promotes the efflux of 5-Fluorouracil (5-FU), leading to the treatment resistance of HCC [17]. In addition, USP22 was able to overcome the effect of Cisplatin on tumors by promoting DNA homologous recombination repair [18]. Interestingly, USP22 is also an essential factor during embryonic development of mice [19]. USP22 contributes to vasculature development during mouse embryogenesis and regulate multiple receptor tyrosine kinase (RTK) pathways including the VEGF pathway [20]. It has been reported that depletion of USP22 suppresses angiogenesis in mouse xenograft model of Non-small cell lung cancer [21], and promotes the effect of sorafenib in HCC [22]. However, the molecular mechanism underlying the effects of USP22 on angiogenesis is still needed to be clarified.

VEGFA plays a crucial role in tumor angiogenesis by binding to its specific receptor VEGFRs in endothelial cells. It has been reported that *VEGFA* is one of the most genomic amplification gene in HCC [23], and over-expression of VEGFA is closely related to HCC progression and rapid recurrence [24, 25]. Recent studies have shown that overexpression of VEGFA activates a variety of oncogenic signaling pathways in HCC-derived cells such as PI3K/Akt, p38 MAPK, PKC, and ERK1/2 in an autocrine–paracrine manner, inducing cancer cell proliferation, metastasis, and vasculogenic mimicry (VM) [25–29].VM describes plasticity of aggressive cancer cells forming abnormal vessel networks by deformation and matrix remodeling [30, 31]. It has been identified as one of the leading cause for the failure of anti-angiogenesis therapy in malignant tumors [30, 32, 33]. In addition, excessive expression of VEGFA inhibits T cells cytotoxicity in HCC microenvironment resulting in tumor immunosuppression, while sorafenib could not reverse this [34]. What’s more, HCC-associated endothelial cells release VEGF-enriched exosomes to overcome the effects of sorafenib and promote tumor angiogenesis [35]. Thus, the identification of the novel co-regulators involved in modulation of *VEGFA* transcription would be important for providing various potential strategy for HCC therapy.

ZEB1 as a transcription factor, has been reported to participate in a variety of signaling pathways and regulates the malignant proliferation, invasion, and metastasis in HCC [36–39]. Ectopic expression of ZEB1 enhances *VEGFA* mRNA expression and promotes angiogenesis, VM formation, and anti-angiogenic therapy resistance in breast cancer, prostate cancer, or colorectal cancer [40–44]. Moreover, in bevacizumab and sorafenib resistant tumor cells, the expression of ZEB1 is increased, and tumors show stronger invasion and metastasis ability [39, 44], suggesting that ZEB1 may play an important role in the process of resistance to anti-angiogenesis therapy. Thus, analysis of novel co-regulator for modulation of ZEB1 action may provide the effective strategy for anti-angiogenic therapy resistance in HCC.

In this study, our results have demonstrated that USP22 up-regulates ZEB1-mediated *VEGFA* transcription. USP22 is recruited to ZEB1-binding elements on the promoter of *VEGFA*, participating in histone H2B deubiquitination. Importantly, USP22 maintains the stability of ZEB1 through its deubiquitinase activity. USP22 promotes Vascular Mimicry (VM) formation, and angiogenesis in HCC. Our results suggest that USP22 acting as a novel co-activator of ZEB1 may contribute to anti-angiogenic therapy resistance in HCC.

## Results

### USP22 is increased with the enhancement of HCC stage, and expression of USP22 is positively correlated with that of VEGFA in HCC

It has been reported that USP22 plays an important role in the biological process of various tumors. However, more biological function of USP22 on HCC remains to be elucidated. To determine the role of USP22 in HCC, we firstly analyzed the clinical data by UALCAN (http://ualcan.path.uab.edu/index.html) which based on The Cancer Genome Atlas (TCGA) and Clinical Proteomic Tumor Analysis Consortium (CPTAC) clinical database. The results showed that mRNA or protein expression of USP22 in HCC samples was significantly higher than those in normal liver tissues (Supplementary Fig. S1A, B). Furthermore, the analyzed data showed that the expression of USP22 was gradually increased with the enhancement of HCC stage (Supplementary Fig. S1C, Table 1). In addition, according to publicly available data (*n* = 340) in The Human Protein Atlas (https://www.proteinatlas.org/), high expression of USP22 was significantly associated with poor clinical outcome in HCC patients (Supplementary Fig. S1D).

**Table 1:** Relationship between USP22 expression and Pathologic Stage in HCC.

Characteristics		Expression of USP22		*P*-value^a^
	Cases (*n* = 339)	Low (*n* = 265)	High (*n* = 74)	
Age (y)				
<60	159	111	48	0.011
≥60	180	154	26	
Gender				
Male	231	181	50	0.905
Female	108	84	24	
Pathologic Stage				
I	169	145	24	0.002
II	83	61	22	
III	83	55	28	
IV	4	4	0	

^a^*χ*^2^ test.

**Fig. 1:**
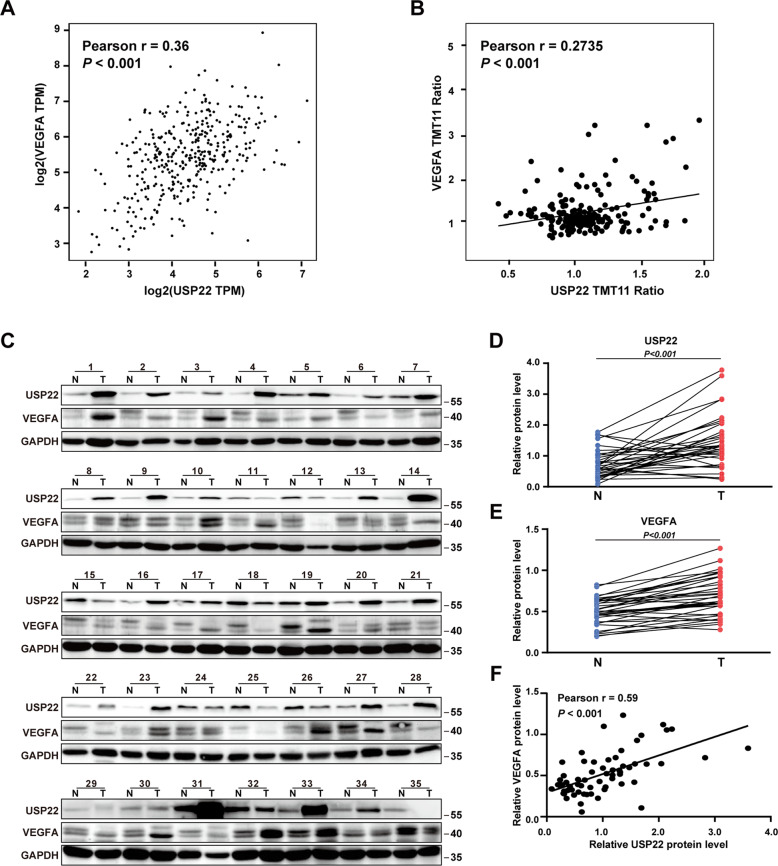
USP22 is positively correlated with VEGFA in HCC. **A**, **B** The mRNA or protein expression of USP22 is positively correlated with that of VEGFA. The data was downloaded from GEPIA (**A**) or CPTAC (**B**). **C** Evaluation of the indicated proteins in 35 clinical HCC samples (T) and the matched adjacent non-cancerous tissues (N) by western blotting. **D**, **E** The protein expression of USP22 or VEGFA in HCC samples is higher than that in the matched adjacent non-cancerous tissues. Data from (**C**) were quantified by densitometry, with GAPDH as the reference. **F** The protein expression of USP22 is positively correlated with VEGFA in 35 pairs of HCC samples and the matched adjacent noncancerous tissues. Pearson r indicates the degree of correlation. *P* < 0.05 is considered statistically significant.

In order to determine whether USP22 is associated with VEGFA expression, a pair-wise gene expression correlation analysis for given sets of TCGA expression data using GEPIA (http://gepia.cancer-pku.cn/index.html) was performed. The results showed a positive correlation between the mRNA level of USP22 and that of VEGFA in HCC samples (Fig. 1A). In addition, the protein expression data given by CPTAC database suggest that the protein level of USP22 was also positively correlated with that of VEGFA in HCC samples (Fig. 1B). We thus examined the expression of USP22 and VEGFA in 35 pairs of HCC samples and the matched adjacent noncancerous tissues by western blotting. The results demonstrated that USP22 or VEGFA expression was significantly higher in HCC samples than that in the matched adjacent noncancerous tissues (Fig. 1C–E). Significant correlation between the protein expression of USP22 and that of VEGFA was observed (Fig. 1F). These data suggested that there was a positive relationship between USP22 and VEGFA. We next plot the gene expression correlation of USP22 with other regulators in VEGF pathway, like VEGFR1, VEGFR2, PI3K, PKCα, IP3 and MEK in GEPIA database. The results showed that USP22 was positively correlated with the expression of these genes, with the *Pearson r* ranging from 0.19 to 0.54 (Supplementary Fig. S2A).

### USP22 increases ZEB1-induced *VEGFA* transcription in HCC cells

**Fig. 2:**
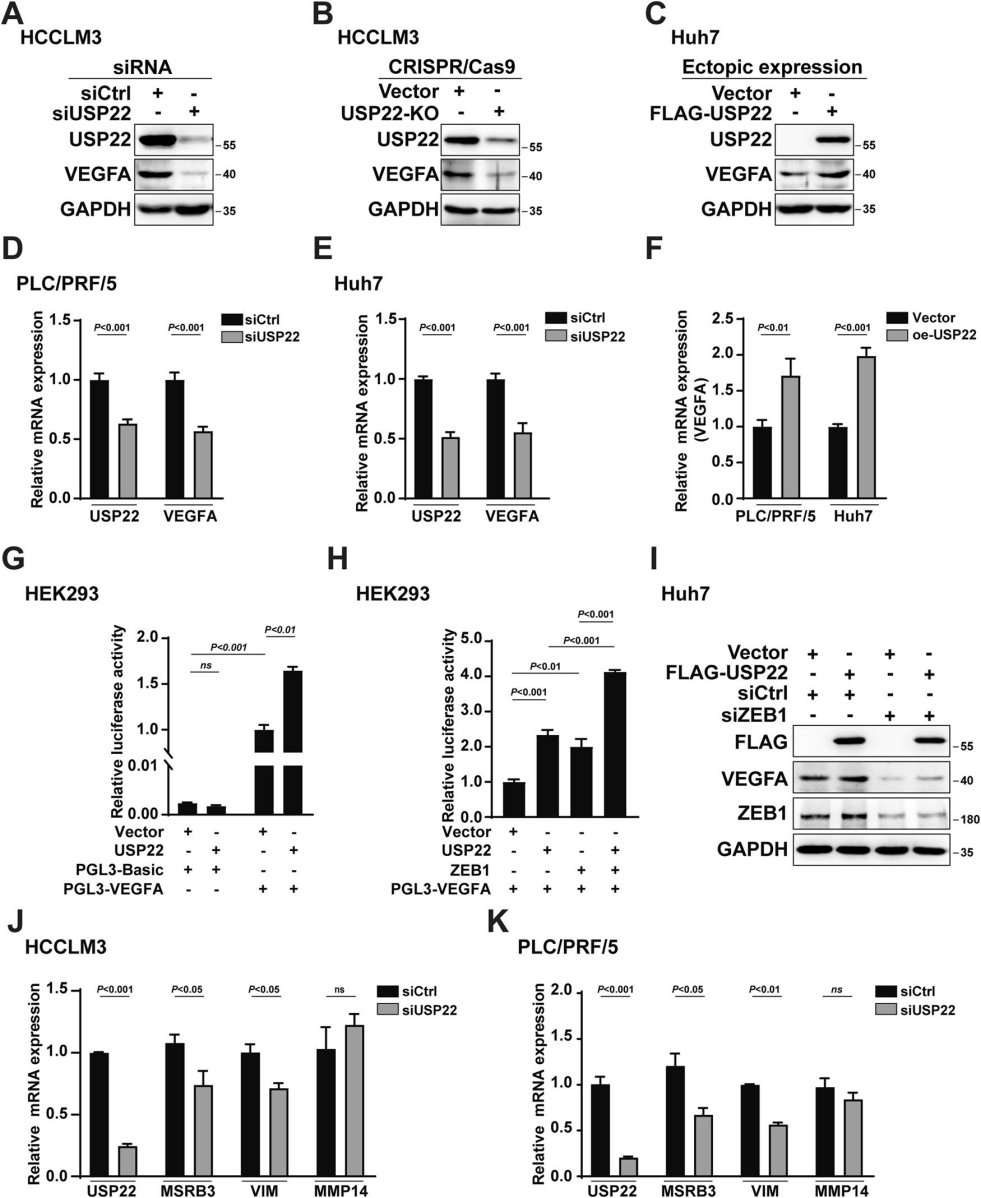
USP22 increases ZEB1-induced *VEGFA* transcription in HCC cells. **A**–**C** The effects of USP22 on protein expression of VEGFA in HCCLM3 or Huh7 cells with GAPDH as the reference. **D**, **E** Evaluation of mRNA expression of *VEGFA* in USP22 knockdown PLC/PRF/5 or Huh7 cells. **F** Evaluation of mRNA expression of *VEGFA* in USP22 overexpressed PLC/PRF/5 or Huh7 cells. **G** USP22 enhances *VEGFA* transcription. HEK293 cells were transfected with PGL3-Basic or PGL3-VEGFA to evaluate the effect of USP22 on the transcription of *VEGFA*. Cells were lysed and assayed using the dual-luciferase reporter assay system. **H** USP22 and ZEB1 co-upregulate *VEGFA* transcription. HEK293 cells were transfected with PGL3-VEGFA together with the indicated expression plasmids. Cells were lysed and assayed using the dual-luciferase reporter assay system. **I** USP22 lost its regulatory effect on VEGFA protein expression when ZEB1 was knocked down. FLAG-USP22 or Vector was transfected with or without ZEB1 siRNA into Huh7 cells. **J**, **K** Evaluation of mRNA expression of three ZEB1 target gene in USP22 knockdown HCCLM3 or PLC/PRF/5 cells.

To further confirm the effect of USP22 on VEGFA expression, either siRNA against USP22 (siUSP22), CRISPR/Cas9 USP22-KO plasmids or USP22 expression plasmids (oe-USP22) was transfected into HCC-derived cells. Western blotting results showed that the protein expression of VEGFA was decreased by knockdown of USP22, while the protein expression of VEGFA was upregulated by overexpression of USP22 (Fig. 2A–C). In addition, quantitative Realtime PCR (qPCR) were further performed. The results demonstrated that the transcription of *VEGFA* was downregulated by USP22 deletion, in contrast, ectopic expression of USP22 increased *VEGFA* transcription (Fig. 2D–F). These results suggested that USP22 may act as a co-activator of *VEGFA* transcription. To conform this conclusion, we further generated a luciferase reporter plasmid containing *VEGFA* promoter sequences. Dual luciferase assay results showed that USP22 significantly upregulated *VEGFA* transcription (Fig. 2G). As an important transcription factor, ZEB1 is known to upregulate *VEGFA* transcription [40, 41]. To detect the effect of USP22 on ZEB1-mediated *VEGFA* transcription, USP22 and ZEB1 expression plasmids were co-transfected into HEK293 cells for luciferase assay as indicated, and the data showed that USP22 enhanced ZEB1-mediated *VEGFA* transcription (Fig. 2H). Western blotting experiments showed that the upregulation of VEGFA by USP22 was inhibited in the absence of ZEB1 (Fig. 2I), this suggested that the up-regulation of *VEGFA* transcription by USP22 was partly dependent on ZEB1 expression. The knockdown efficiency of ZEB1 was shown in the Supplementary Fig. S2B. In addition, RNA-seq analysis for clinical HCC samples from GEPIA database also suggests that USP22 mRNA expression was positively correlated with those of ZEB1 downstream target genes, including *MSRB3, MMP14 and VIM* (vimentin) (Supplementary Fig. S2C). Realtime PCR was performed in HCCLM3 or PLC/PRF/5 cells. The results showed that USP22 knockdown significantly decreased the mRNA levels of *MSRB3* and *VIM*, but had no obvious effect on that of *MMP14* (Fig. 2J, K). Taken together, these results suggest that USP22 may act as a co-activator of ZEB1-mediated *VEGFA* transcription.

### USP22 interacts with ZEB1, and USP22/ZEB1 is recruited to the ZEB1-binding elements of VEGFA promoter region in HCC cell lines

**Fig. 3:**
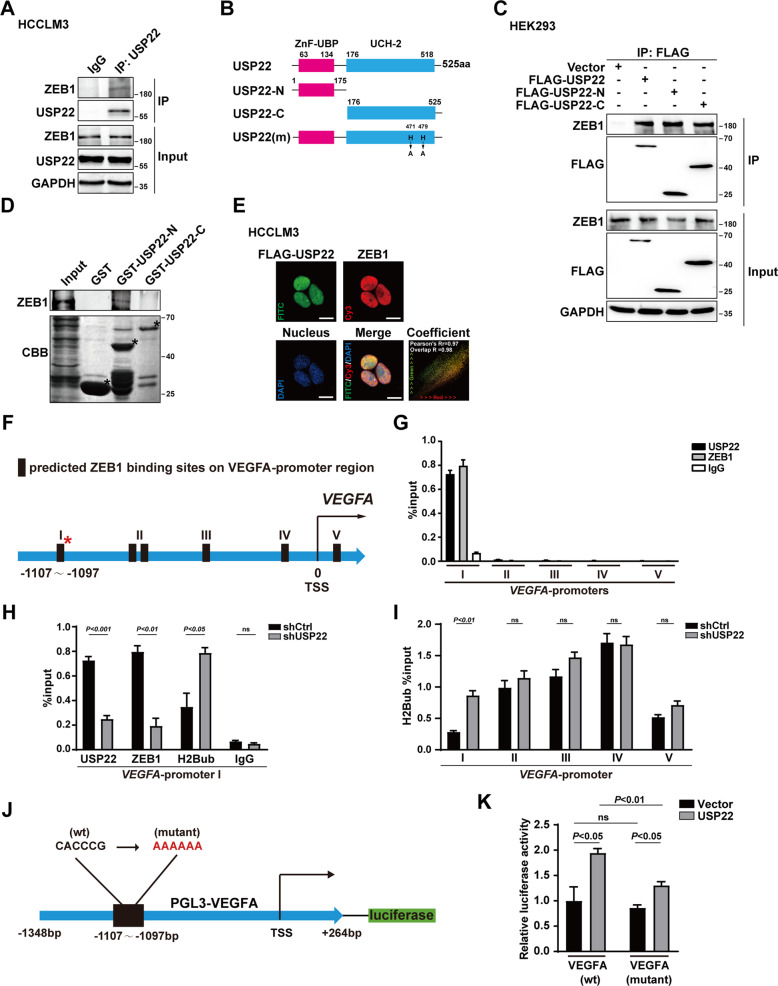
USP22 interacts with ZEB1, and USP22/ZEB1 is recruited to the ZEB1-binding elements of *VEGFA* promoter region in HCC cell lines. **A** The endogenous interaction between USP22 and ZEB1 in HCCLM3 cells verified by Co-Immunoprecipitation. **B** Diagram of full-length (FL) and truncated mutants of USP22. **C** Exogenous USP22 or its truncated mutants interact with ZEB1 in HEK293 cells. **D** Identification of binding domains in USP22 for ZEB1 interaction. ZEB1 protein was synthesized by transcription and translation kit in vitro. Bound proteins were analyzed by western blot. GST and GST- USP22 deletion mutants were stained by Coomassie brilliant blue staining. *, position of GST and GST- USP22 deletion mutants. **E** The Immunofluorescence confocal experiments were used to identify the localizations of FLAG-USP22 (green) and ZEB1 (red) in HCCLM3 cells. HCCLM3 cell was transfected with FLAG-USP22 expression plasmid for 24 h before harvested. Nucleus was stained by DAPI (blue), scale bars, 15 μm. The colocation coefficient was analyzed and calculated by Image Pro Plus software. **F** Schematic diagram of the predicted ZEB1 binding sites on *VEGFA* promoter region. **G** USP22 were recruited to ZEB1-binding elements in *VEGFA*. **H** USP22 knockdown reduced the recruitment of ZEB1 and accumulation of levels of H2Bub on ZEB1-binding site at *VEGFA*-promoter I. The DNA fragments were amplified by qPCR with the primers indicated in Supplementary data (Table S3). **I** Effects of USP22 on the ubiquitination level of histone H2B near the five predicted ZEB1 binding sites. **J** Schematic diagram of PGL3-VEGFA mutant (ZEB1 binding site mutation) luciferase assay plasmid, CACCCG was replaced by AAAAAA. **K** USP22 failed to upregulate transcription of *VEGFA* without wild type ZEB1 binding site in dual luciferase assay. In histogram, the bars represent mean ± SD (*n* ≥ 3), *P* < 0.05 is considered statistically significant.

We thus turned to ask whether USP22 could interact with ZEB1 in HCC cell lines. Co-immunoprecipitation (Co-IP) experiments were performed in HCC cells. The results demonstrated that the endogenous USP22 associates with ZEB1 in HCCLM3 and Huh7 cell lines (Fig. 3A and Supplementary Fig. S3A, B). In addition, HEK-293 cells were transfected with USP22 and ZEB1 expression plasmids as indicated and the interaction between USP22 and ZEB1 was conformed in Co-IP experiment (Supplementary Fig. S3C). To map the region within USP22 that mediates its interaction with ZEB1, truncated mutants of USP22 were generated (Fig. 3B). To our surprise, both N and C terminus of USP22 associates with ZEB1 (Fig. 3C). Further, we constructed the truncated GST-USP22 plasmids with the same structure as Fig. 3B. The results showed that only the N terminal of USP22 directly binds to ZEB1 (Fig. 3D), while the C terminal of USP22 may indirectly bind to ZEB1. Moreover, the results of immunofluorescence experiments showed that transfected FLAG-USP22 (FITC, green) and endogenous ZEB1(Cy3, red) were distributed in the nucleus in HCC-derived cells or HEK293 cells (Fig. 3E and Supplementary Fig. S3D). Taken together, these data demonstrated that USP22 physically associates with ZEB1 in HCC cells.

To examine whether USP22 was recruited to ZEB1-binding elements of *VEGFA*, we identified five potential ZEB1 binding sites on the promoter region of *VEGFA* based on the JASPAR database (Supplementary Fig. S4A) [45], and we named them as *VEGFA*-promoter I/II/III/IV/V (Fig. 3F). Chromatin immunoprecipitation (ChIP) assay was performed in HCCLM3 cells. The results demonstrated that USP22 or ZEB1 was recruited to *VEGFA*-promoter I dominantly (Fig. 3G). To examine whether USP22 affected the histone H2Bub or ZEB1 recruitment to the *VEGFA*-promoter I region, we first constructed HCCLM3 cell lines with stable knockdown of USP22 (shUSP22) by lentivirus infection, a scramble shRNA as a control (shCtrl). The knockdown efficiency was confirmed by qPCR and western blotting (Supplementary Fig. S4B, C). ChIP assays were further performed with shUSP22 HCCLM3 cells. The results showed that USP22 deletion reduced the recruitment of ZEB1, and induced accumulation of histone H2Bub at *VEGFA*-promoter I region (Fig. 3H). Our results showed that knockdown of USP22 only increased the histone H2B ubiquitination level at ZEB1 binding site I on *VEGFA* promoter, while had no obvious effect on the histone ubiquitination level at other sites (Fig. 3I). We examined the relationship between USP22 expression and total histone H2B ubiquitination in HCCLM3 cells. Our results showed that overexpression of USP22 had no significant effect on total histone H2B ubiquitination in HCCLM3 cells (Supplementary Fig. S4D).

To further determine whether *VEGFA*-promoter I region is required for the transcription activity of *VEGFA*-promoter, we constructed mutant *VEGFA* promoter reporter plasmids with AAAAA sequence instead of CACCCG sequence at *VEGFA*-promoter I region as indicated. (Fig. 3J). Dual luciferase assays results showed that the upregulation effect induced by USP22 was significantly reduced on mutant *VEGFA* promoter reporter (Fig. 3K). We also constructed different truncated VEGFA promoter reporter plasmids according to the predicted ZEB1 binding sites in Fig. 3F. Our results showed that the up-regulatory effect of USP22 on ZEB1-induced *VEGFA* transcription was significantly reduced after loss of ZEB1 binding site I in the presence of USP22 (Supplementary Fig. S4E, F). These results suggest that USP22 or ZEB1 is recruited to *VEGFA*-promoter I region (−1107 to −1097), thereby reducing the level of histone H2Bub to enhance ZEB1 mediated *VEGFA* transcription.

### USP22 maintains ZEB1 stability via triggering deubiquitination of ZEB1

**Fig. 4:**
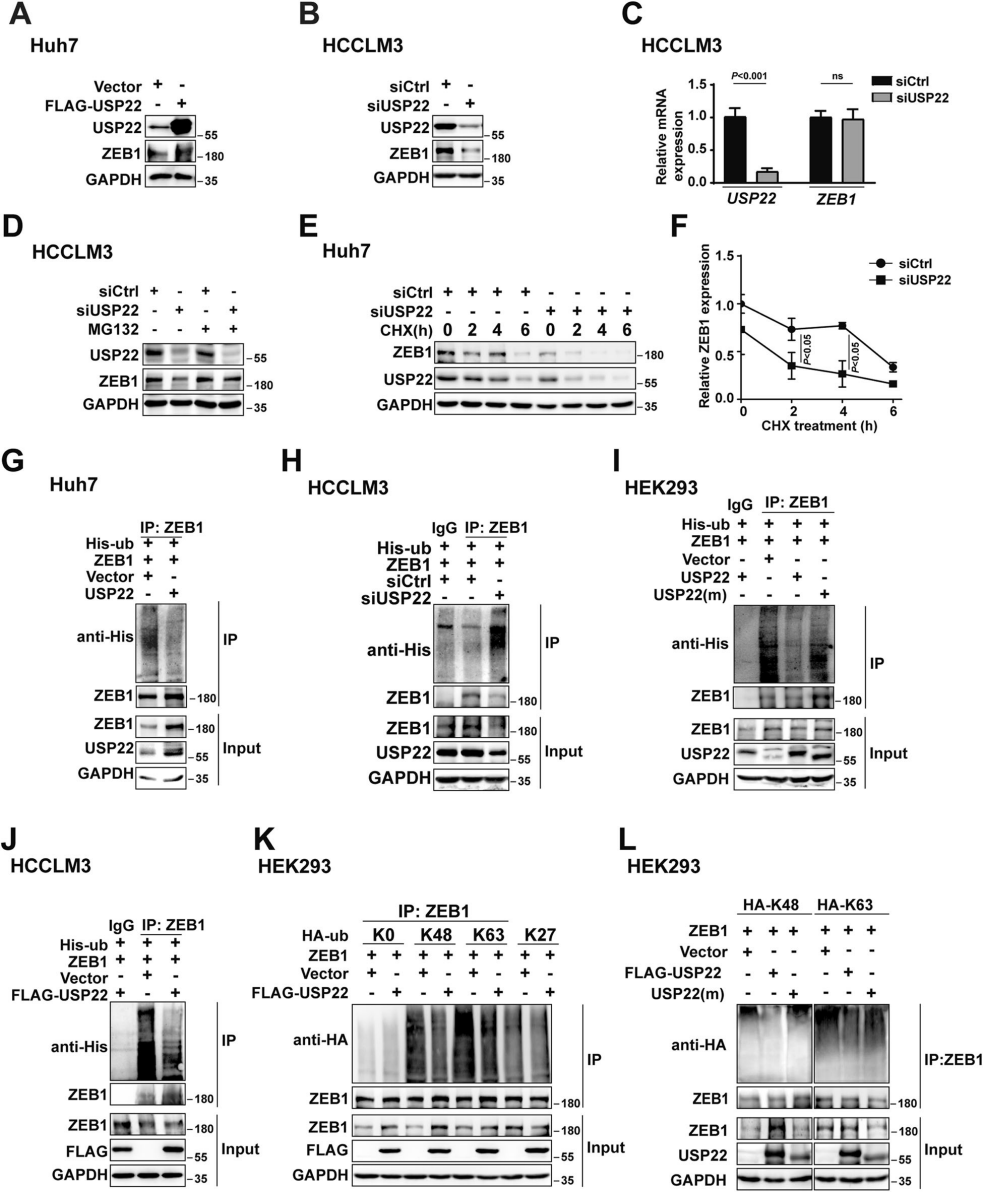
USP22 maintains ZEB1 stability by triggering deubiquitination of ZEB1. **A**, **B** USP22 deletion reduced ZEB1 protein expression while ectopic expression of USP22 upregulated that in HCC-derived cell lines in western blotting experiments. **C** USP22 had no obvious effect on mRNA expression of ZEB1 in qPCR experiments, the bars represent mean ± SD (*n* ≥ 3), *P* < 0.05 is considered statistically significant. **D** Western blotting analysis of ZEB1 expression in wild-type and USP22 knockdown Huh7 cells treated with MG132 (10 μM) for 8 h as indicated. **E** Depletion of USP22 decreases endogenous ZEB1 protein stability in HCCLM3 cells. Cells were treated with 100 μM cycloheximide (CHX) as indicated. **F** Gray values of ZEB1 were calculated and presented in a line chart with statistical analysis. **G** Ectopic expression of USP22 decreased the ubiquitination of ZEB1. Immunoprecipitation of ubiquitinated proteins from Huh7 cell extracts upon overexpression of USP22. Protein was harvested after MG132 (5 μM) treatment for 3 h and ubiquitinated ZEB1 species were detected by western blotting with anti-His. **H** Ubiquitination of ZEB1 was upregulated by USP22 deletion. HCCLM3 cells were transfected with USP22 siRNA. Cells were immunoprecipitated with ZEB1 and immunoblotted with anti-His. **I** The ubiquitination level of ZEB1 could not be reduced by deubiquitinase inactive mutant USP22(m). Cells were immunoprecipitated with ZEB1 and immunoblotted with anti-His. HEK293 cells were transfected with expression plasmid encoding ZEB1 and His-ubiquitin together with wild type USP22 or USP22(m). **J**–**L** Denaturing ubiquitination assays were performed to detect the effects of USP22 on ubiquitination levels and types of ZEB1. HCCLM3 cells were transfected with ZEB1 and FLAG-USP22 together with His-tagged ubiquitin (**J**). HEK293 cells were transfected with ZEB1 and FLAG-USP22 or USP22(m) together with HA-tagged ubiquitin mutants, including K0 (lysineless), K48-, K63-, and K27-linked ubiquitin as indicated (**K**, **L**). The cells were treated with MG132 (5 μM) before collected. The cell lysate was immunoprecipitated with anti-ZEB1 and immunoblotted with anti-His or anti-HA.

Having established that USP22 associates with ZEB1, and USP22/ZEB1 is recruited to the promoter of *VEGFA* in HCC cells, we thus turn to examine whether USP22 participates in maintenance of ZEB1 stability. Western blotting and qPCR experiments were performed. Our results showed that ectopic expression of USP22 increased ZEB1 protein expression and USP22 deletion reduced ZEB1 protein expression, while USP22 had no obvious effect on mRNA expression of ZEB1 in HCC-derived cell lines (Fig. 4A–C). Ectopic expression of USP22 did not increase the protein expression of ZEB1 in the presence of ubiquitin proteasome inhibitor MG132. Reduction of ZEB1 caused by knockdown of USP22 was also prevented by MG132, on the other hand, USP22 depletion accelerated ZEB1 degradation with the treatment of cycloheximide (CHX), which is protein synthesis inhibitor, in HCC-derived cells (Fig. 4D–F and Supplementary Fig. S5A). These results suggest that USP22 is involved in maintaining the stability of ZEB1. Immunoprecipitation (IP)-based ubiquitination assays were further performed to determine the effects of USP22 on ZEB1 deubiquitination. The results demonstrated that the ubiquitination level of ZEB1 was significantly decreased by ectopic expression of USP22 in HCCLM3 and Huh7 cells (Fig. 4G and Supplementary Fig. S5B). USP22 depletion or USP22 deubiquitinase inactive mutant USP22 (m) enhanced the ubiquitination level of ZEB1 (Fig. 4H, I). Denaturing ubiquitination assay was used to examine the deubiquitination effect of USP22 on ZEB1, which could exclude the interference of other proteins. All the lysates were denatured prior to antibody recognition. The results showed that overexpression of USP22 also reduced the ubiquitination level of ZEB1 in HCCLM3 cells (Fig. 4J). Taken together, our data indicate that USP22 maintains ZEB1 stability by triggering the deubiquitination of ZEB1.

In order to determine the types of poly-ubiquitination processes influenced by USP22, HA-tagged Lysine 48 (K48)-, K63- and K27-linked ubiquitin chains were used in denaturing ubiquitination assay in HEK293 cells. The results demonstrated that K48- and K63- linked ubiquitination of ZEB1 were notably reduced under ectopic USP22 expression while K27-linked ubiquitination was not (Fig. 4K). In contrast to wild-type USP22, inactive deubiquitinase mutant USP22 (m) failed to cleave K48- and K63-linked polyubiquitination on ZEB1 (Fig. 4L). Taken together, these results indicate that USP22 participates in the maintenance of ZEB1 stability through at least, if not all, K48- and K63-linkage.

### USP22 promotes HCC-derived cell growth/invasion/Vascular Mimicry (VM) formation and angiogenesis

**Fig. 5:**
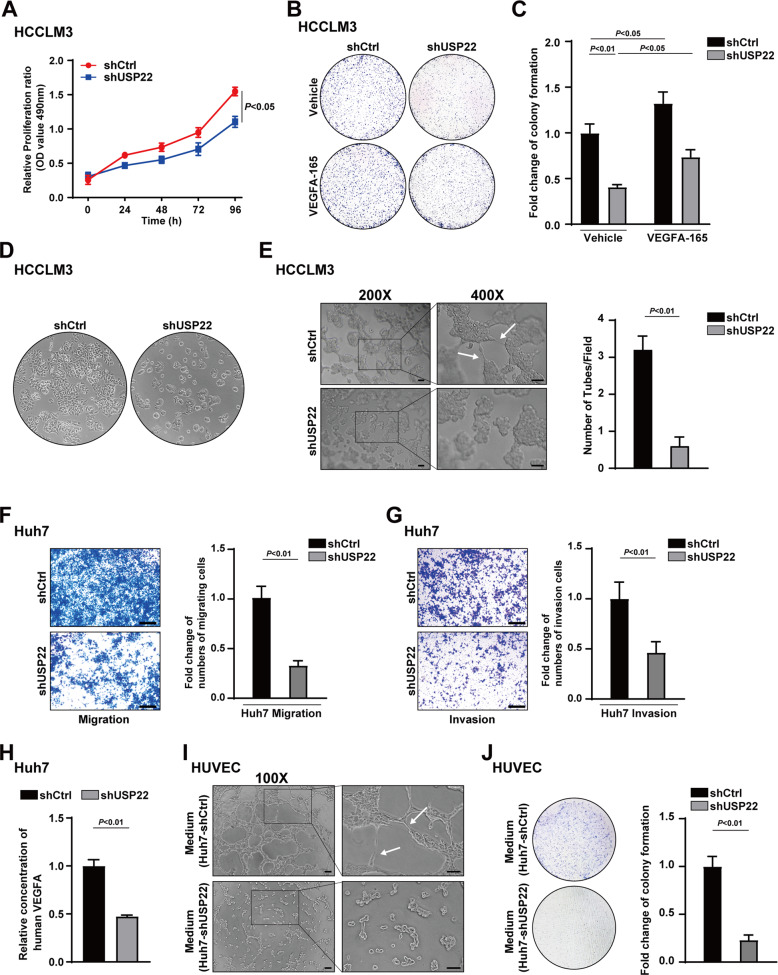
USP22 promotes HCC-derived cell growth/invasion/Vascular Mimicry (VM) formation and angiogenesis. **A** Knockdown of USP22 inhibited HCCLM3 proliferation. The absorbance of HCCLM3 cells carrying shCtrl or shUSP22 added MTS reagent in indicated time was measured at 490 nm. **B**, **C** Effects of USP22 on the cell growth in HCCLM3 cells were photographed in the colony formation assay. VEGFA-165 stands for recombinant human VEGFA protein. **D** The morphology of HCCLM3 cells changed with shUSP22 was photographed under microscope. **E** The left panel shows tube formation pictures of the HCCLM3 cells. Scale bars, 200 μm. The right panel is a quantification plot of the number of tubes in each visual field in the left. **F**, **G** Effect of USP22 on cell migration and invasion in Huh7 cells. Cells were planted in the transwell chamber coated with Matrigel (**G**) or without Matrigel (**F**) for 24 h. Scale bars, 250 μm. **H** ELISA assay was used to verify the effect of USP22 on VEGFA secretion from Huh7 cells. **I** Tube formation of HUVEC cells were photographed under microscope. Conditioned medium collected from shCtrl or shUSP22 Huh7 cells were applied to culture HUVEC cells on the Matrigel for 24 h. **J** Colony formation experiments were used to determine the effect of USP22 on the cell growth of HUVEC cells. Scale bars, 250 μm. Histogram data were shown as mean ± SD, *P* < 0.05 is considered statistically significant.

To further investigate the effect of USP22 on HCC progression, we first generated Huh7 cells and PLC/PRF/5 cells with stable knockdown of USP22 (shUSP22) by lentivirus infection, a scramble shRNA as a control (shCtrl) as indicated. The USP22 knockdown efficiency was conformed using western blotting experiment (Supplementary Fig. S6A, B). MTS assay and cell colony formation experiments were performed to determine the effects of USP22 on cell proliferation of HCC cells. The results showed that the cell proliferation was significantly inhibited with USP22 depletion (Fig. 5A and Supplementary Fig. S6C). The inhibited cell proliferation was partially recovered by treatment with recombinant human protein VEGFA-165, a predominant isoform with properties closely corresponding to native VEGFA [46] (Fig. 5B, C). Our data indicate that USP22 promotes cell proliferation in HCC cells and the effect of USP22 on cell growth is partially related to VEGFA.

We observed that USP22 knockdown resulted in morphological atrophy of HCC cells and enhanced cell-to-cell adhesion to be difficult to form association between cell colonies (Fig. 5D). These results suggest that USP22 may participate in matrix remodeling or movement of HCC cells. Vasculogenic mimicry (VM) describes plasticity of aggressive cancer cells forming de novo vascular networks by deformation and matrix remodeling [30, 31]. Thus, tube formation assay was performed to examine the effect of USP22 on VM formation induced by HCCLM3 cells. The results showed that USP22 deletion remarkably inhibited tubular network formation (Fig. 5E). These results suggest that the expression of USP22 may promote VM formation in HCC-derived cell lines.

Next, we turn to determine the effect of USP22 on migratory and invasive behaviors of HCC cells, transwell assays were performed in shUSP22 Huh7 or shUSP22 PLC/PRF/5 cells. Our data demonstrate that USP22 deletion inhibits the migration and invasion of HCC cells (Fig. 5F, G and Supplementary Fig. S6D).

To further investigate the potential effect of USP22 on angiogenesis, we first performed ELISA assay to detect the VEGFA level in the culture supernatant of Huh7 cells. The results showed that USP22 deletion inhibited VEGFA secretion (Fig. 5H). Conditioned medium collected from shUSP22 or shCtrl Huh7 cells were used to perform HUVEC tube formation assay in vitro. The results demonstrated that intercellular connections between HUVEC cells were reduced in conditioned medium derived from shUSP22 Huh7 cells (Fig. 5I). HUVEC colony formation assay cultured in conditioned medium as indicated showed that the proliferation of HUVEC was also decreased in conditioned medium derived from shUSP22 Huh7 cells (Fig. 5J). These data suggest that USP22 promotes angiogenesis in HCC partially related to VEGFA expression. Taken together, our data demonstrate that USP22 is involved in HCC proliferation, invasion, VM formation, and angiogenesis.

### USP22 promotes HCC cell growth and VM formation in mice Xenograft

**Fig. 6:**
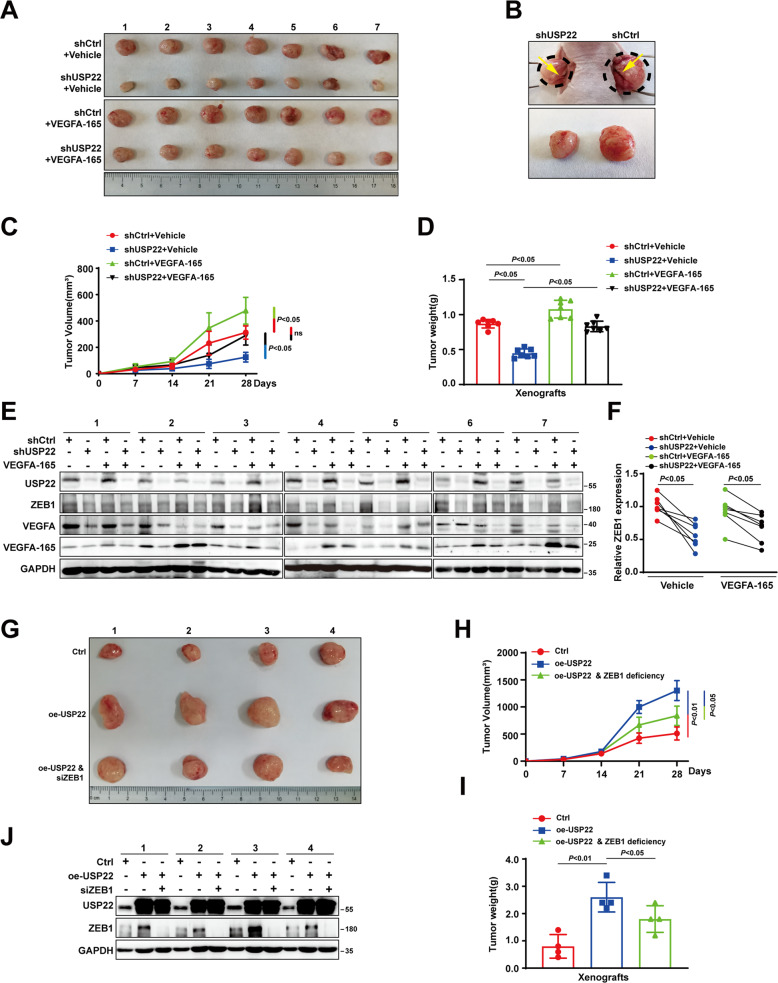
USP22 promotes HCC progression in mice. **A** Pictures showed all the xenograft tumors formed by HCCLM3 cells in different groups. VEGFA-165 stands for recombinant human protein VEGFA-165. **B** The image closely up displays the tumors in situ, with yellow arrows indicating blood vessels forming on the tumor surface. **C** Growth curves drawn by tumor volume of xenograft tumors in the USP22 knockdown group and the control group at indicated time. **D** Tumor weight of xenografts in indicated groups were shown. **E** Protein levels of USP22, VEGFA and ZEB1 in every xenograft tumor were detected by western blotting. GAPDH was used as a control. **F** The protein expression of ZEB1 from (**E**) was quantified by densitometry, with GAPDH as the reference. *P* < 0.05 is considered statistically significant. **G** Pictures showed all the xenograft tumors formed by HCCLM3 cells in different groups. **H** Growth curves drawn by tumor volume of xenograft tumors in the USP22 overexpressing, USP22 overexpressing with ZEB1 deletion, and the control group at indicated time. **I** Tumor weight of xenografts in indicated groups were shown. **J** Protein levels of USP22, and ZEB1 in every xenograft tumor were detected by western blotting. GAPDH was used as a control.

To examine the function of USP22 in HCC growth in mice, tumor growth analysis in a mouse xenograft model was performed. As shown in Fig. 6A–D, tumors from shUSP22 HCCLM3 cells were smaller in size, and grew at a lower rate than those from shCtrl cells. The blood vessels formed in shUSP22 tumors were thinner than those in control. Injection of VEGFA-165 partially restored the tumor growth inhibition induced by USP22 knockdown. We then examined the expression of USP22, VEGFA, and ZEB1 in those xenograft tumors. The results demonstrated USP22 knockdown reduced mRNA or protein expression of VEGFA. While USP22 depletion decreased ZEB1 protein, not mRNA level (Fig. 6E, F and Supplementary Fig. S7A). In addition, the expression of USP22 was also positively correlated with that of VEGFA in xenograft tumor (Supplementary Fig. S7B). In order to test the role of ZEB1 in our model, lentivirus was used to construct HCC cells with stable overexpression of USP22. HCCLM3 cells in the control group (*n* = 4), the USP22 overexpression group (*n* = 8) were injected subcutaneously into BALB/c nude mice for xenograft experiments. Two weeks later, when the tumor size was approximately 100 mm^3^, mice carrying USP22-overexpressing tumors were randomly divided into two groups (*n* = 4): siRNA of ZEB1 (7.5 μg) was injected into the tumor, and the other group was injected with the same amount of negative control every 72 h for another 2 weeks. The results showed that, overexpression of USP22 significantly promoted the growth of HCC, and knockdown of ZEB1 inhibited the effect of USP22 on tumor growth (Fig. 6G–J), suggesting that ZEB1 was involved in the development of HCC, and the promotion of HCC growth by USP22 was partially related to ZEB1.

**Fig. 7:**
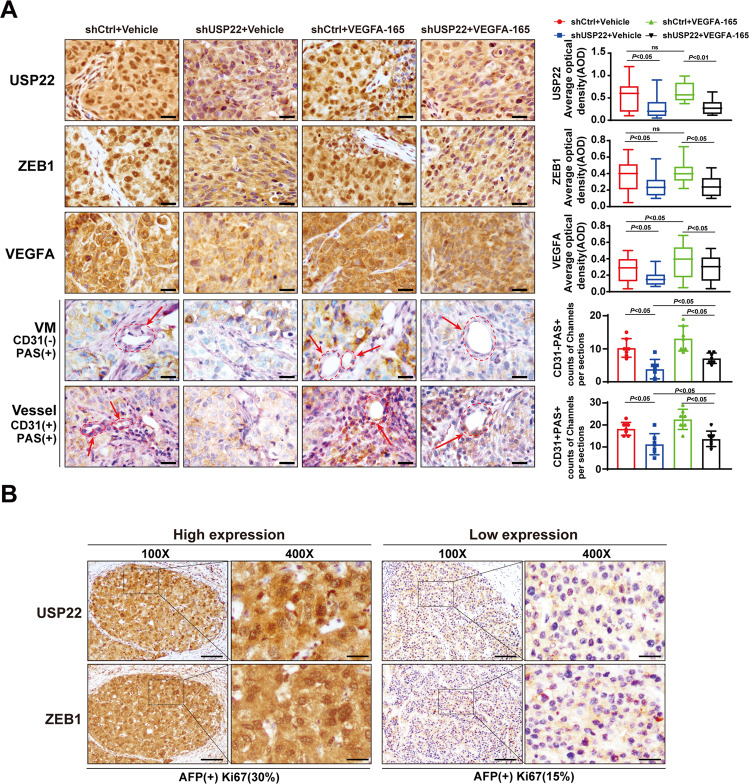
Knockdown of USP22 reduced VM formation in xenograft tumors. **A** Expression of USP22, ZEB1 and VEGFA in xenograft tumors (Fig. 6A) were detected by IHC assays; CD31/PAS double staining was used to evaluate the appearance of vasculogenic mimicry (PAS + CD31-) and vessels (PAS + and CD31 + ) in the shUSP22 and shCtrl groups. VEGFA-165 stands for recombinant human protein VEGFA-165. Scale bars, 50μm. Average optical density (AOD) was used to evaluate the relative expression levels of USP22, ZEB1 and VEGFA. Counts of channels per sections was used to evaluate the effect of USP22 on the formation of vascular mimicry and angiogenesis within tumors. **B** The representative images of expression of USP22 and ZEB1 in immunohistochemical staining of human HCC pathological sections. The expression of specific marker from the HCC pathological data showed that, the tumor with high USP22 and ZEB1 expression on the left, AFP (+), Ki67(30%); the tumor with low USP22 and ZEB1 expression on the right, AFP (+), Ki67 (15%). Scale bars, 200 μm.

In addition, the results from immunohistochemistry showed that USP22 depletion decreased USP22, ZEB1, or VEGFA expression in xenograft tumors. Periodic acid–Schiff (PAS) staining is commonly used to indicate the presence of glycogen or polysaccharide (purplish red) to access the possibility vascular infiltration in the tumor. The CD31 and PAS dual staining is applied to distinguish the matrix-rich morphological pattern of VM [29]. VM structure is identified as vascular-like channels that were lined by tumor cells, and displays PAS positive/CD31 negative staining (PAS + CD31-), while the parts stained with PAS + and CD31 + represent endothelial vessels. Our data showed that the number of VM (PAS + CD31- staining) is less in shUSP22 than that in shCtrl tumors, suggesting that knockdown of USP22 may inhibit VM formation (Fig. 7A and Supplementary Fig. S7C). Taken together, our results indicate that USP22 promotes HCC cell growth and VM formation in mice.

Having revealed that USP22 maintains ZEB1 stability, we next examined the expression of USP22 and ZEB1 in 24 pairs of human HCC pathological sections by immunohistochemistry. The results showed that USP22 expression was positively correlated with that of ZEB1 in HCC (Fig. 7B and Supplementary Fig. S7D). According to the data provided by The Human Protein Atalas, HCC patients with high expression of VEGFA or ZEB1 had poor prognosis. In addition, high expression of USP22/VEGFA or USP22/ZEB1 at the same time was positively correlated with poor prognosis (Supplementary Fig. S8). These results suggest that USP22/ZEB1/VEGFA promotes HCC progression.

## Discussion

**Fig. 8:**
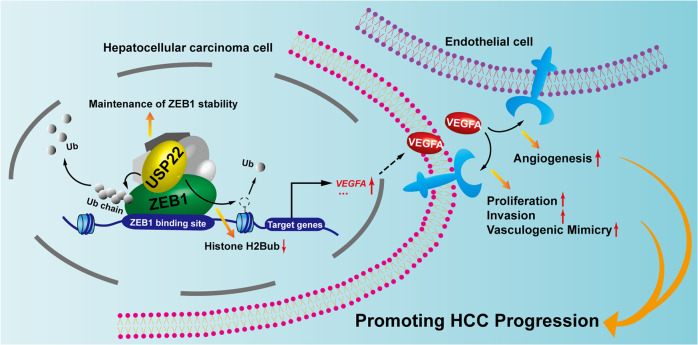
Schematic diagram illustrating the function of USP22 on modulation of ZEB1-induced *VEGFA* transcription and the role of USP22 in promotion of HCC progression. USP22 maintains ZEB1 stability by triggering deubiquitination of ZEB1. USP22/ZEB1 being recruited to the ZEB1 binding elements of *VEGFA* promoter region, inducing the ubiquitination of histone H2B, enhances the transcription of *VEGFA*.This effect ultimately leads to increased expression and secretion of VEGFA, which may accelerate HCC progression.

As one of the most genomic amplification gene in HCC, VEGFA has been recognized as a crucial role in HCC progression, and antiangiogenic agents have been approved for advanced-stage HCC [1, 24]. However, the benefit from these therapies is still transient and unsatisfactory. Identification of novel regulators of VEGFA would provide potential therapeutic targets for anti-angiogenic therapy resistance in HCC. In this study, our data demonstrate that USP22 acts as a ZEB1 co-activator to upregulate ZEB1-induced *VEGFA* transcription. Our results suggest that USP22 participates in promotion of HCC progression, if not all, at least partially via VEGFA expression (Fig. 8).

As an important transcription factor, ZEB1 promotes epithelial-mesenchymal transition (EMT) and distal metastasis in a variety of tumors [47]. It is also known that ZEB1 increases the expression of VEGFA, and plays an important role in promoting angiogenesis, vasculogenic mimicry (VM) and anti-angiogenic therapy resistance [40–42, 44, 48]. Interestingly, previous studies have shown that ZEB1 has dual activity to promote or inhibit gene transcription due to interact with different co-regulators. CtBP interacts with ZEB1 to co-repress the transcription of epithelial genes, such as *CDH1* (E-cadherin). While P300 or P/CAF interacts with ZEB1 to coactivate mesenchymal factors, such as *CDH2* (N-cadherin), *VIM* (Vimentin) [41, 47]. Here, our results have demonstrated that USP22 interacts with ZEB1, and USP22/ZEB1 is recruited to the ZEB1-binding elements of *VEGFA* promoter region and USP22 decrease the accumulation of histone H2Bub. USP22 upregulates ZEB1-mediated *VEGFA* transcription in HCC cell lines. (Figs. 1–3). Our studies indicate that USP22 acting as a histone deubiquitinase participates in deubiquitination of histone H2Bub on the promoter of *VEGFA*, co-activating ZEB1-mediated *VEGFA* transcription.

It has been reported that ubiquitination modification on ZEB1 involved in maintenance of ZEB1 stability plays important roles in tumor progression. Ubiquitination of ZEB1 induced by the E3 ubiquitin ligase CHFR contributes to the sensitivity of TNBC to chemotherapy drugs [49]. The stability of ZEB1 maintained by USP43 and USP51 promotes the proliferation and metastasis of colorectal and breast cancer [50, 51]. USP39 and E3 ligase TRIM26 balance the level of ZEB1 ubiquitination, thereby determining the progression of hepatocellular carcinoma [38]. In our study, we identified USP22 as a novel co-regulator of ZEB1. USP22 maintains the protein stability of ZEB1 by triggering the deubiquitination of K48- and K63-linked polyubiquitin chains on ZEB1 via its deubiquitinase activity (Fig. 4). Our findings here identify that USP22 as a histone deubiquitinase participates in non-histone deubiquitination to maintain ZEB1 stability, thus co-activating ZEB1-mediated transactivation.

Having demonstrated that USP22 is identified as one of the 11 genes to be involved in cancer-related death signatures, and plays a crucial role in the progression of tumor [6, 52]. USP22 positively regulates c-Myc, androgen receptor, and HIF-1α actions to promote breast cancer, prostate cancer, and HCC progression [16, 53, 54]. On the other hand, USP22 deficiency decreases PU.1 stability to promote myeloid leukemia [10]. USP22 accelerates necroptotic cell death in several cancer cells via regulating RIPK3 ubiquitination [55]. These studies suggest that USP22 possesses the implicated biological functions in pro- or anti-tumor effects by catalyzing the different substrates. Here, our data identified that USP22 acts as a potential co-activator of ZEB1 through histone and non-histone modifications to be involved in up-regulation of *VEGFA* transcription in the promotion of HCC progression. It has been reported that ZEB1 or VEGFA plays a driven role in VM and angiogenesis [28, 29, 33, 35, 56, 57]. VM has been identified as one of the leading cause for the failure of anti-angiogenesis therapy in malignant tumors [30, 32, 33]. Our results have demonstrated that USP22 contributes to VM formation and angiogenesis as shown in Figs. 5–7. Importantly, it has been recognized that VEGFA also plays a crucial role in tumor immunosuppression [58–60], and our results suggest that USP22 may participate in tumor immune escape via modulation of VEGFA expression. This interesting study would be further explored.

Taken together, our study has demonstrated that USP22 is a novel co-regulator of ZEB1 to enhance ZEB1-induced *VEGFA* transcription via USP22 deubiquitination activity, thereby promoting tumor growth/invasion/VM formation and angiogenesis in HCC. Our results provide the evidence to support that USP22 plays a driven role in HCC progression and might be a potential therapeutic target for anti-angiogenic therapy resistance in HCC.

## Materials and methods

### Antibodies and plasmids

The antibodies used in this study: anti-USP22 (Proteintech, 55110-1-AP), anti-USP22 (abcam, ab195289), anti-ZEB1 (Proteintech, 21544-1-AP), anti-VEGFA (Proteintech, 66828-1-Ig), anti-CD31 (Proteintech, 11265-1-AP), anti-His-tag (Proteintech, 66005-1-Ig), anti-HA (Shanghai genomics, GNI4110), anti-GAPDH (ABclonal Technology, AC033), anti-Ubiquity-Histone H2B (Lys120) (Cell Signaling Technology, 5546).

The expression plasmids of wild-type human USP22 and mutant USP22 (HH/AA) were described as previous studies [8, 15]. Two truncated mutants of USP22 were cloned into the pcDNA3.1 vector containing a FLAG-tag for Co-IP experiments, or cloned into pGEX-5X-1 vector containing GST-tag for GST pull-down experiments. Full (−1348 to +264) and a series of truncated *VEGFA* promoter sequence were cloned into a PGL3-basic vector for luciferase assay. In the mutated PGL3-VEGFA plasmid, CACCCG was replaced by AAAAAA (−1107 to −1097), while other sequences remained unchanged. The plasmid purchased in this study: ZEB1(Sino Biological, HG17705-UT), CRISPR-Cas9 USP22-KO (Santa Cruz, sc-403660-NIC).

### Cell culture

All cell lines were grown at 37 °C under 5% CO_2_ in 10% fetal bovine serum. HCCLM3, Huh7 and HEK293 cells were cultured in Dulbecco’s Modified Eagle’s Medium (DMEM). PLC/PRF/5 cells were cultured in Minimum Essential Medium (MEM). Human umbilical vein endothelial cell (HUVEC) was cultured in HUVEC medium as previously described [61]. All the cell lines were recently authenticated by PCR-STR analysis.

### siRNA transfection and lentiviral infection, RNA isolation, reverse transcription, and quantitative real-time PCR (qPCR)

For RNA interference (RNAi), the small interfering RNA (siRNA) of USP22 was purchased from Sigma Aldrich and siRNA of ZEB1 was purchased from GenePharma. The sequences of siRNA were listed in Supplementary Table S1. For lentivirus-delivered RNAi, lentiviral productions were purchased from Shanghai GeneChem Company. Total RNA was extracted by RNA isolater (Vazyme). Reverse transcription was performed using HiScript II Q RT SuperMix for qPCR (Vazyme). The cDNAs were quantified by qPCR using ChamQ Universal SYBR qPCR Master Mix (Vazyme) on LightCycler 96 instrument (Roche). Primers used to detect mRNA expression were purchased from Thermo Fisher Scientific and the sequences were shown in Supplementary Table S2.

### Co-Immunoprecipitation (Co-IP), western blotting, immunofluorescence assay

For Co-Immunoprecipitation (Co-IP) in HCCLM3 and Huh7 cells, the whole cell lysates were extracted and the equal protein amounts were immunoprecipitated with USP22 antibody or IgG. Then Protein G Sepharose 4 Fast Flow incubated. The immune complexes were detected by western blotting using indicated antibody. For Co-IP in HEK293 cells, the expression plasmids of ZEB1and USP22 or Vector were transfected using the jetPRIME reagent (Polyplus) for 24 h, and then the whole cell lysates were extracted. For ubiquitination Co-IP, His- or HA-tag ubiquitin were co-transfected with ZEB1 and USP22 or Vector for 24 h. Cells were treated with 10 μM MG132 for 3 h before harvested. The extracts were immunoprecipitated with ZEB1 antibody. For denaturing ubiquitination assay, the cells were collected from 5–10 cell volumes of denaturing cell lysis buffer and boiled for 10 min. The denaturing buffer was diluted with cell lysis buffer and the primary antibody was added for immunoprecipitation experiments, and the subsequent steps were the same as for the Co-IP experiments.

For immunofluorescence assay, HEK293 cells were transfected with FLAG-USP22 and ZEB1 plasmids, while HCCLM3 and Huh7 cells were transfected with FLAG-USP22 in advance for 12 h. Then HEK293, HCCLM3 and Huh7 Cells were fixed in 4% paraformaldehyde and permeabilized with 0.1% TritonX-100. All the cells were incubated with anti-USP22 and anti-ZEB1 antibodies overnight at 4 °C and subsequently FITC or Cy3-conjugated secondary antibody (Jackson Immunoresearch Laboratories Inc, Cat#JAC-711-095-152, JAC-711-165-152). Nuclei were stained with DAPI (Roche). Respective images were taken under confocal microscopy (Nikon).

### Luciferase reporter assay

Cells were co-transfected with the listed constructs and pRL (Renilla luciferase control). The cell lysates were detected by the dual luciferase reporter assay system (Promega) as specification described.

### Chromatin Immunoprecipitation (ChIP) assay

ChIP assays were performed according to previously described protocols [15]. Immunoprecipitation of sonicated chromatin solutions was conducted by incubated with indicated antibody at 4 °C overnight, subsequently incubated with protein A-sepharose for 4 h. With phenol-chloroform, DNA fragments were extracted, and then precipitated in ethanol. The purified DNA was analyzed by qPCR. Results were shown as percentage of input chromatin. Primer sequences for ZEB1 binding sites of *VEGFA* were listed in Supplementary Table S3.

### ELISA assay

Huh7 cells infected with lentivirus carrying shUSP22 or shCtrl were pre-cultured in fresh medium for 24 h, and VEGFA levels in cell-culture supernatant were determined using an ELISA kit (mlbio, ml057663) according to the manufacturer’s instructions.

### MTS assay, colony formation assay, and tube formation assay

2 × 10^3^ cells were plated in 96-well plates and measured using MTS assay (Promega) with the absorbance at 490 nm. 1 × 10^3^ cells were plated in 35 mm dish for 7 days, then cells were fixed with 4% paraformaldehyde and stained with Coomassie brilliant blue dye for colony formation assay. VEGFA-165 was purchased from AbMole (M9413). 1 × 10^4^ HCCLM3 or HUVEC cells were planted in the Matrigel (BD Biosciences)-coated 96 plates for tube formation assay as previously described with minor modification [35]. Conditioned medium collected from shUSP22 or shCtrl Huh7 cells as previously described were used to perform HUVEC tube formation assay in vitro [61]. The growth status of the cells was photographed under phase-contrast microscopy. Each experiment was performed in triplicate.

### Transwell migration and invasion assay

6 × 10^4^ cells were dispersed into serum-free medium and then separately added to each transwell chamber (Corning, NY). The lower part of the chambers was placed in 10% serum medium. The chambers with Matrigel-coated were for cell invasion, and those without Matrigel-coated for cell migration. Then the cells were fixed with 95% ethanol and stained with Coomassie brilliant blue dye or Crystal Violet.

### Xenograft tumor growth

HCCLM3 cells infected with lentivirus carrying shUSP22 or shCtrl were separately injected into left (shUSP22) or right(shCtrl) flanks of 5 weeks old male BALB/c nude mice (*n* = 14). After fixed tumors formed, half of the mice were injected with human recombinant protein VEGFA-165 around each tumor and the other half were injected with the same amount of normal saline for 4 weeks. Tumor volume (cubic millimeters) was calculated as volume = (short diameter)^2^ × (long diameter)/2 [22]. All animal experiments have complied with the ARRIVE guidelines. No animals suffered unnecessarily hurt at any stage of an experiment. HCCLM3 cells infected with lentivirus carrying oe-USP22 or Ctrl were injected subcutaneously into BALB/c nude mice for Control group (*n* = 4) and USP22 overexpression group (*n* = 8). Two weeks later, when the tumor size was approximately 100 mm^3^, mice carrying USP22-overexpressing tumors were randomly divided into two groups (*n* = 4): siRNA of ZEB1 (7.5 μg) was injected into the tumor, and the other group was injected with the same amount of negative control every 72 h for another 2 weeks. Tumor-bearing mice were killed with the policy of the humane treatment of animals. All procedures of animal experiments have been carried out in compliance with ethical regulations approved by the Animal Ethics Committee of China Medical University.

### Collection of clinical HCC tissue samples, immunohistochemical (IHC) and Periodic Acid-Schiff (PAS) stain

Human HCC tissues and corresponding adjacent tissues were obtained from the first affiliated hospital China Medical University. The informed consents of the patients were obtained. IHC experiment was performed as previously described [15]. The reagent for IHC stain was purchased from OriGene. The signals were visualized with diaminobenzidine (DAB) and the nuclei were counterstained with hematoxylin.

According to the instructions, for Periodic Acid-Schiff (PAS) staining, the tissue sections were oxidized after dewaxing to water, then stained in Schiff staining solution, and the nuclei were stained with hematoxylin; For PAS/CD31 double staining, the tissue sections were placed in periodate acid for oxidation after chromogenic reaction by DAB, and the following experimental procedures were the same as for PAS staining.

The average optical density (AOD, IOD/Area) obtained by Image Pro Plus image processing software were used to evaluate the indicated protein expression.

### Statistics

All statistical analysis was performed using the SPSS statistical (26.0) software program. Student’s two-tailed *t*-test was used for the determination of statistical relevance between groups. Survival curves were estimated by the Kaplan-Meier method. The average optical density was used as the IHC scoring criterion. *P*-value less than 0.05 was considered statistically significant.

## Supplementary information

cdd-author-contribution-form

aj-checklist

Supplementary Figure

Supplementary Figure Legends

Supplementary Table

Original Western blots

## Data Availability

All data generated or analyzed during this study are included in this published article and its Supplementary information files. The data for all bioinformatics analyses in this article were derived from public databases, including UALCAN, GEPIA, CPTAC, and The Human Protein Atlas. The other datasets used and analyzed during the current study are available from the corresponding author on reasonable request.
