# Supplementary material for: USP22 upregulates ZEB1-mediated VEGFA transcription in hepatocellular carcinoma
Source: Cell Death Dis. 2023 Mar 11;14(3):194. doi: 10.1038/s41419-023-05699-y (PMC10008583; doi:10.1038/s41419-023-05699-y)
Supplement: Supplementary file 1 — cdd-author-contribution-form [file 41419_2023_5699_MOESM1_ESM.pdf]

DECLARATION OF CONTRIBUTIONS TO ARTICLE

ADMC

Manuscript Number: CDDIS-22-3830-T

Journal Name: *Cell Death & Disease* (the 'Journal')

Proposed Title of the Contribution:

USP22 upregulates ZEB1-mediated VEGFA transcription in hepatocellular carcinoma (the 'Contribution)

Author(s):

Kai Zeng, Weiwen Xie, Chunyu Wang, Shengli Wang, Wei Liu, Yingjie Su, Lin Lin, Renlong Zou, Ge Sun, Baosheng Zhou, Manlin Wang, Ruina Luan, Yu Bai, Yunlong Huo, Shigeaki Kato, Xinping Zhong, Yue Zhao (the 'Authors')

For all *CDD* articles, each person named as an author in the published version must be able to show he or she has contributed substantially to the article.

Authorship credit should be based on 1) substantial contributions to conception and design, acquisition of data, or analysis and interpretation of data; 2) drafting the article or revising it critically for important intellectual content; and 3) final approval of the version to be published. Authors should meet conditions 1, 2 and 3.

Any person who cannot be shown to have made a substantial contribution to the article cannot be listed as an author in the final version. The name of any person who is deemed to have made a minor contribution can, however, appear in the Acknowledgments section of the article.

Please complete the table below to indicate the contributions of all named authors to the manuscript.

Author Full Name:	Specification of Contribution to the Manuscript:
Yue Zhao	conception and design the project, drafting the article
Kai Zeng	drafting the article, acquisition of data, analysis and interpretation of data
Weiwen Xie	drafting the article, acquisition of data, analysis and interpretation of data
Chunyu Wang	acquisition of data and drafting the article
Shengli Wang	drafting the article
Wei Liu	acquisition of data
Yingjie Su	acquisition of data
Lin Lin	acquisition of data
Renlong Zou	acquisition of data
Ge Sun	acquisition of data
Baosheng Zhou	acquisition of data
Manlin Wang	acquisition of data
Ruina Luan	acquisition of data

Author Full Name:	Specification of Contribution to the Manuscript:
Yu Bai	acquisition of data
Yunlong Huo	acquisition of data
Shigeaki Kato	revised it critically for important intellectual content and final approval of the version to be published
Xinping Zhong	revised it critically for important intellectual content

Please complete the table below to indicate the contributions of all named authors to the figures.

Figure 1:
Kai Zeng, Weiwen Xie

Figure 2:
Kai Zeng, Weiwen Xie

Figure 3:
Kai Zeng, Weiwen Xie

Figure 4:
Kai Zeng

Figure 5:
Kai Zeng, Weiwen Xie, Ge Sun, Manlin Wang, Ruina Luan, Yu Bai

Figure 6:
Kai Zeng, Wei Liu, Yingjie Su, Baosheng Zhou

Figure 7:
Kai Zeng, Chunyu Wang, Lin Lin, Renlong Zou

Figure 8:
Kai Zeng

Signed for and on behalf of the Author(s):	Print Name:	Date:
Yue Zhao	Yue Zhao	February 17, 2023
