# Supplementary material for: USP22 upregulates ZEB1-mediated VEGFA transcription in hepatocellular carcinoma
Source: Cell Death Dis. 2023 Mar 11;14(3):194. doi: 10.1038/s41419-023-05699-y (PMC10008583; doi:10.1038/s41419-023-05699-y)

Supplementary Figure 1

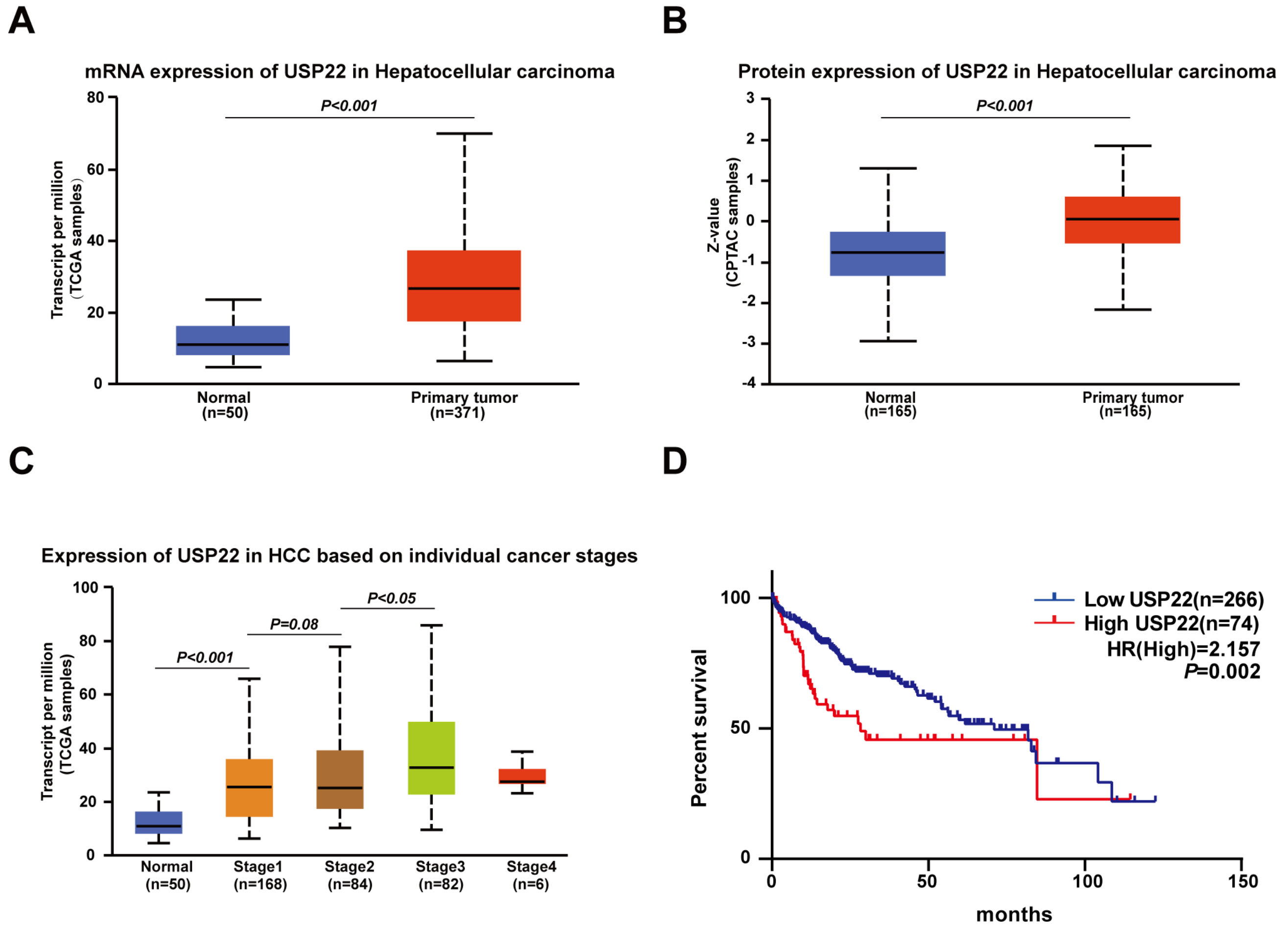

Supplementary Figure 2

A

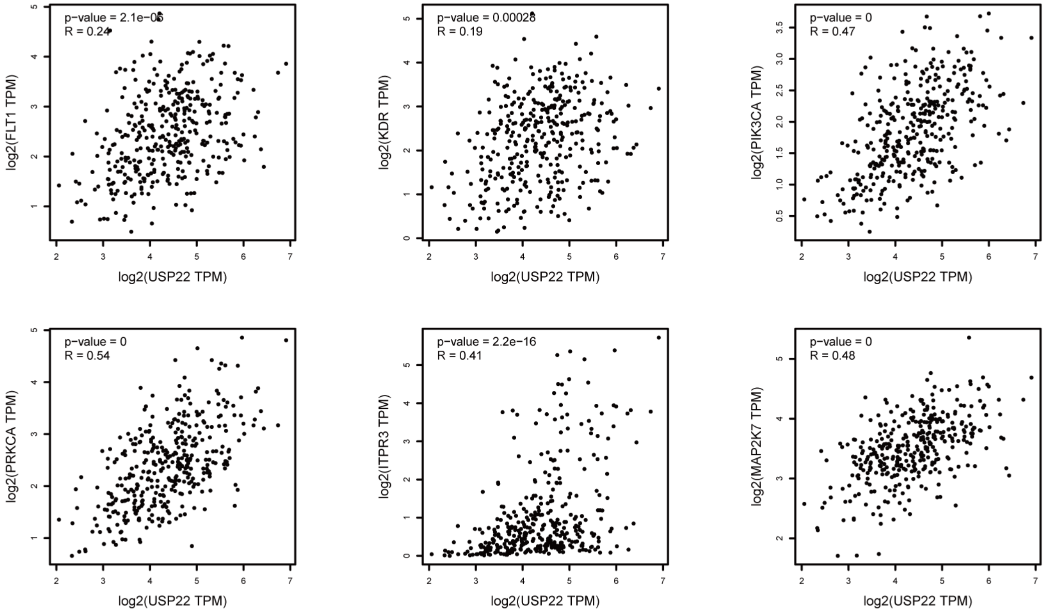

B

Huh7

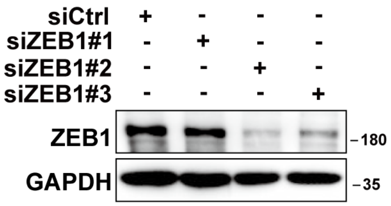

C

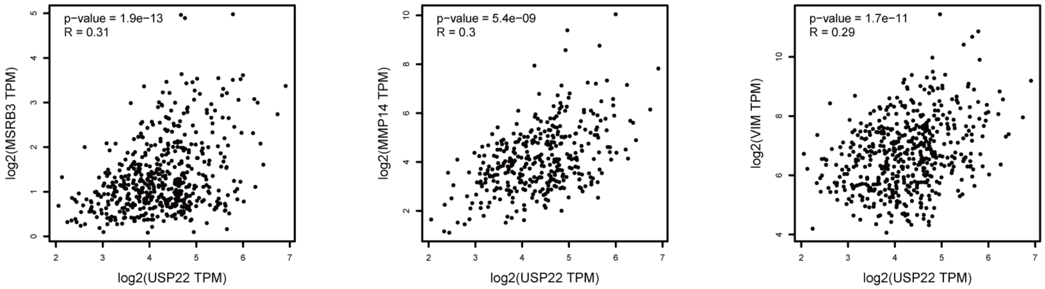

Supplementary Figure 3

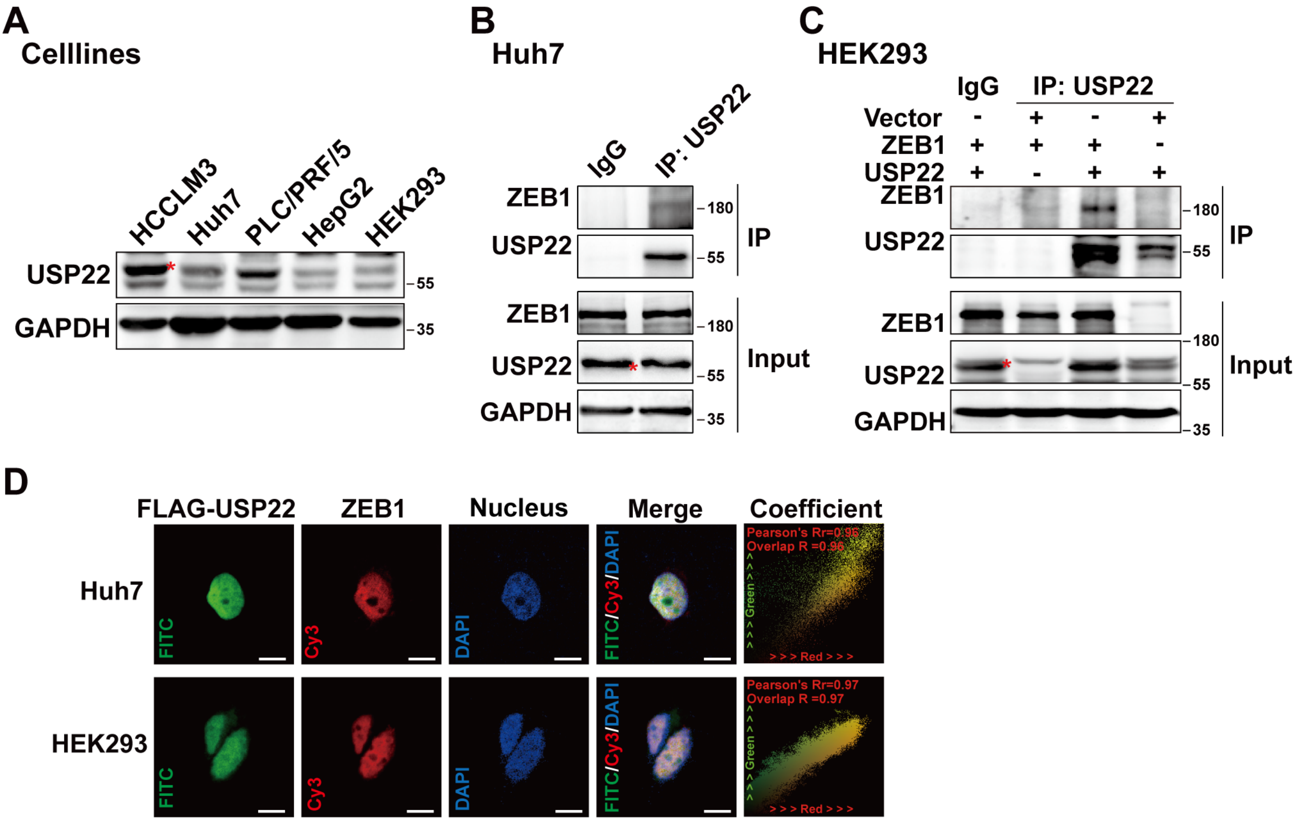

Supplementary Figure 4

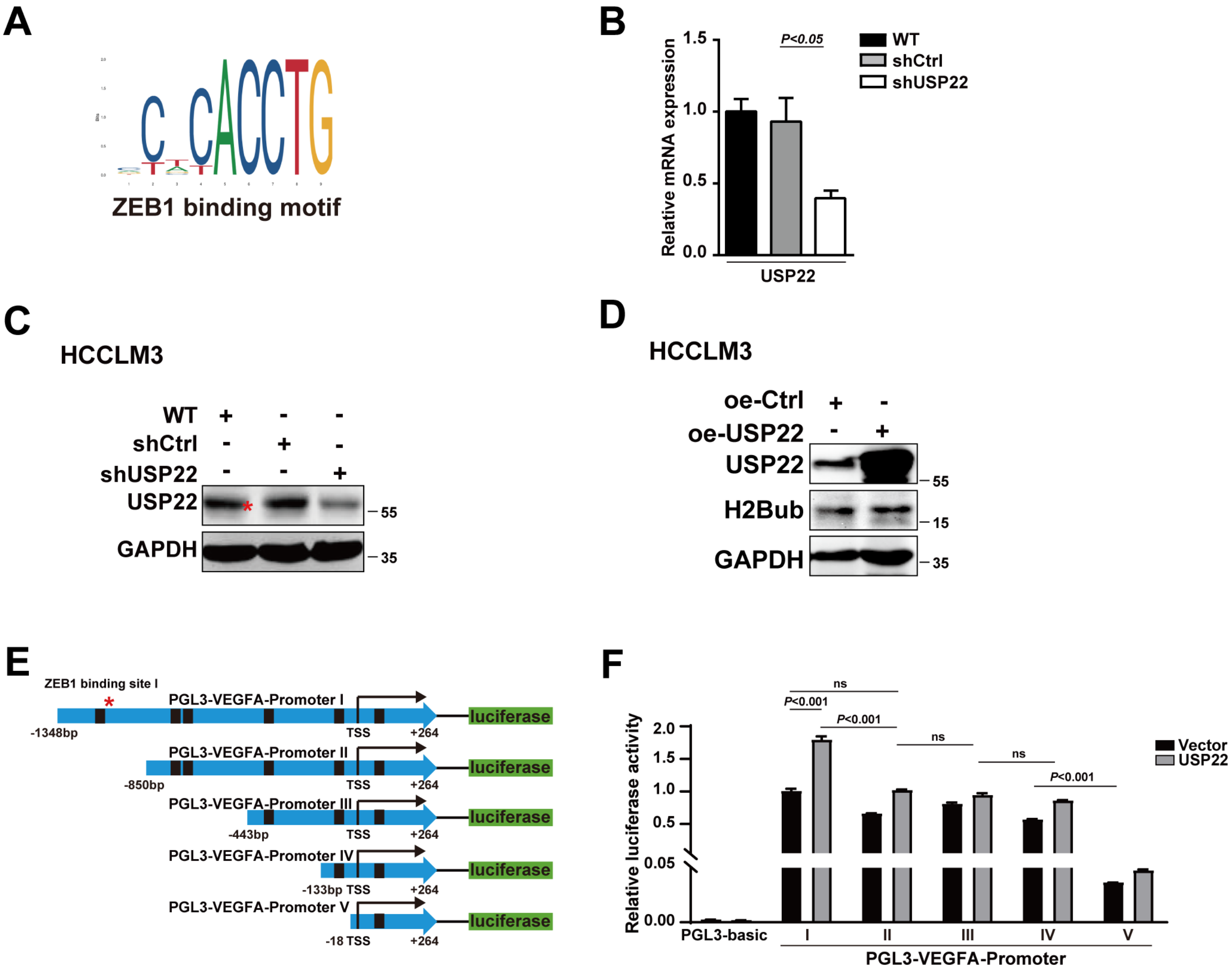

Supplementary Figure 5

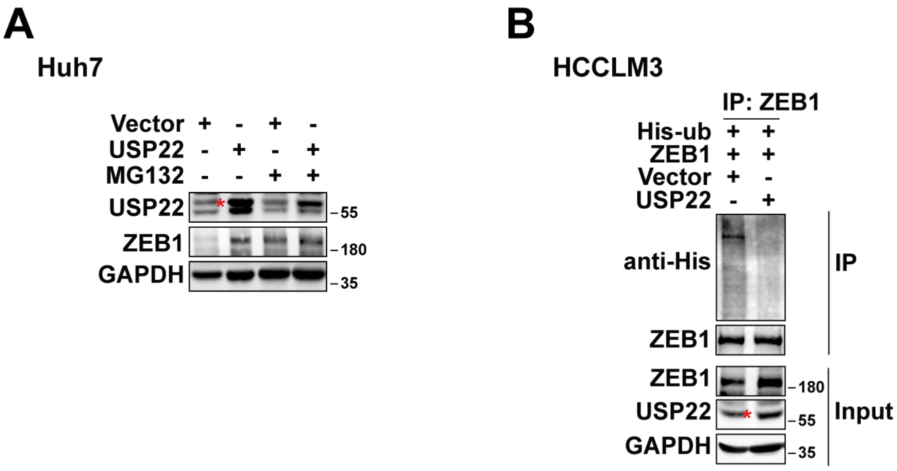

Supplementary Figure 6

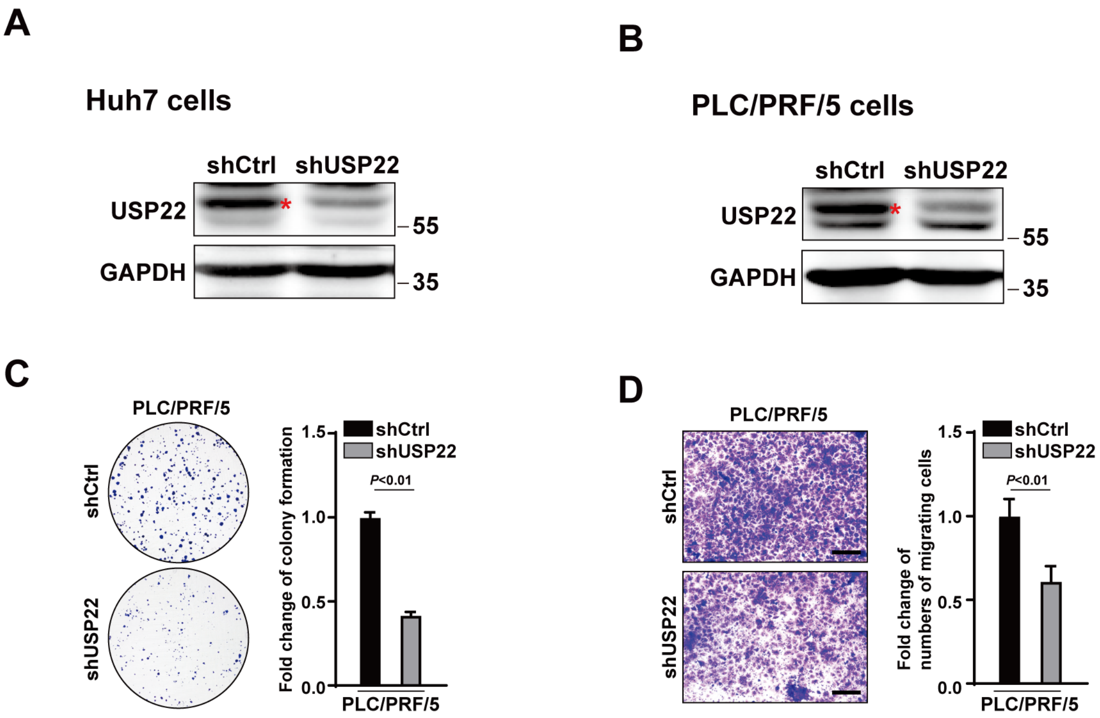

Supplementary Figure 7

A

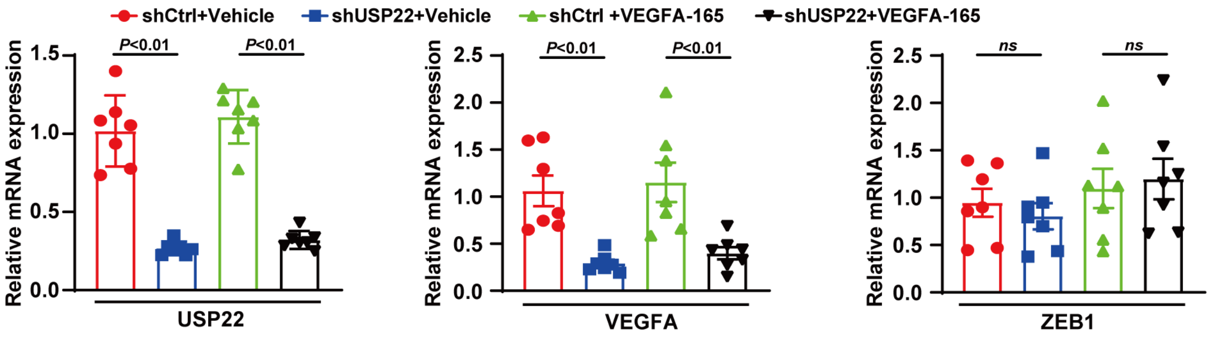

B

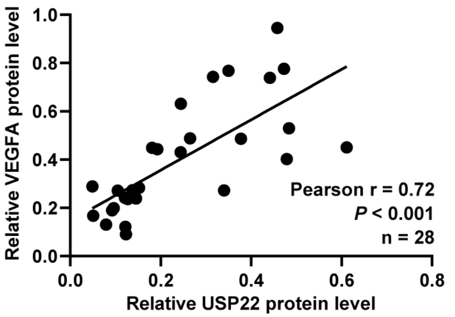

C

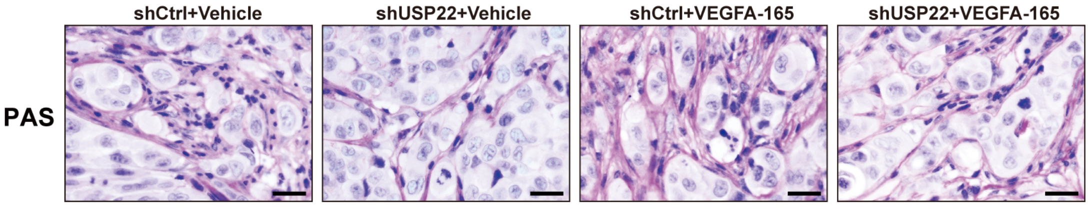

D

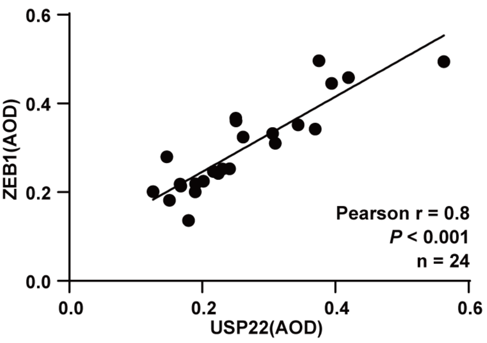

Supplementary Figure 8

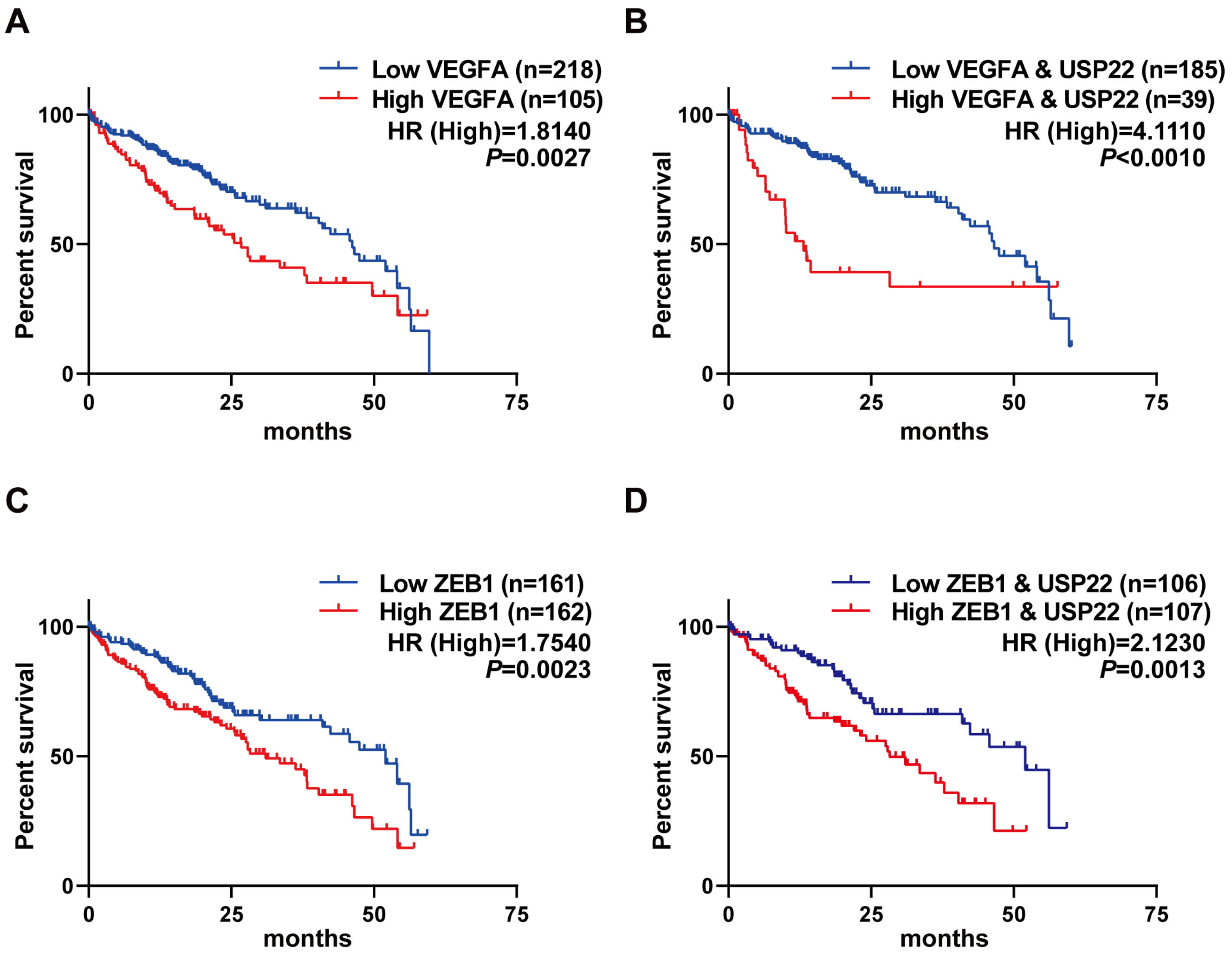

Supplement: Supplementary file 3 — Supplementary Figure [file 41419_2023_5699_MOESM3_ESM.pdf]
