# Supplementary material for: USP22 upregulates ZEB1-mediated VEGFA transcription in hepatocellular carcinoma
Source: Cell Death Dis. 2023 Mar 11;14(3):194. doi: 10.1038/s41419-023-05699-y (PMC10008583; doi:10.1038/s41419-023-05699-y)
Supplement: Supplementary file 4 — Supplementary Figure Legends [file 41419_2023_5699_MOESM4_ESM.docx]

**SUPPLEMENTARY FIGURE LEGENDS**

**Supplementary Fig. S1 USP22 is highly expressed in hepatocellular carcinoma. A** The mRNA expression of USP22 is higher in HCC tumors than in normal. **B** The protein expression of USP22 is higher in HCC tumors than in normal. **C** The mRNA expression of *USP22* correlates with pathological stages of HCC. **D** The overall survival of HCC patients with high expression of *USP22* is poor, which is evaluated by Kaplan-Meier survival analyses. All above data were downloaded from UALCAN and The Human Protein Atlas database. *P* < 0.05 was considered statistically signiﬁcant.

**Supplementary Fig. S2 USP22 was positively correlated with the mRNA of several regulators in VEGF signaling pathway and downstream target genes of ZEB1. A**

GEPIA database shows positive correlation between USP22 and VEGFR1, VEGFR2, PI3K, PKCα, IP3 and MEK. **B** The siRNA knockdown efficiency of ZEB1 was detected by western blotting experiments with GAPDH as the reference. **C** GEPIA database shows positive correlation between USP22 and the ZEB1 downstream genes, including MSRB3, VIM and MMP14. mediated by in. In histogram, the bars represent mean ± SD (n = 3), P < 0.05 is considered statistically signiﬁcant.

**Supplementary Fig. S3 USP22 interacts with ZEB1. A** Western blotting shows the expression of USP22 in several hepatocellular cancer cells. * in western blotting was placed to indicate the position of USP22. **B** The endogenous interaction between USP22 and ZEB1 in Huh7 cells verified by Co-Immunoprecipitation. **C** The exogenous association between USP22 and ZEB1 in HEK-293 cells by Co-IP. HEK-293 cells were transfected with the indicated expressing plasmids. * in western blotting was placed to indicate the position of USP22. **D** Subcellular localizations of the FLAG-USP22 and ZEB1. Huh7 and HEK293 cells were co-transfected with plasmids expressing FLAG-USP22 and ZEB1. Cells were fixed and stained with antibody against FLAG (green) or ZEB1 (red). DAPI was used to visualize the nucleus (blue). Merged images were shown as indicated. Scale bar, 15μm. The co-location coefficient was analyzed and calculated by Image Pro Plus software.

**Supplementary Fig. S4 Effects of USP22 on total ubiquitination of histone H2B and on a series of *VEGFA* promoter truncation transcription. A** The classic binding sequence of ZEB1 given by JASPAR website. **B** qPCR assay shown the knockdown efficiency of USP22 in HCCLM3 cells. **C** Western blotting assay demonstrated knockdown efficiency of USP22 in HCCLM3 cells. **D** Western blotting assay demonstrated the effect of USP22 on total ubiquitination of histone H2B in HCCLM3 cells. **E** Structural diagram of a series of truncated VEGFA promoter reporter plasmids. **F** The transcriptional regulation of USP22 on a series of truncated VEGFA promoters was detected by luciferase assay. Equal amounts of ZEB1 and pRL plasmid were co-transfected into Huh7 cells with different reporter plasmids for Luciferase assay to detect the effect of ZEB1 mediated *VEGFA* transcription in different reporters carrying truncated deletion of ZEB1 binding sites with or without USP22. The bars represent mean ± SD (n ≥ 3), *P* < 0.05 is considered statistically signiﬁcant.

**Supplementary Fig. S5 USP22 maintains ZEB1 stability by triggering deubiquitination of ZEB1. A** Western blotting analysis of the effect of ectopic USP22 expression on ZEB1 expression in Huh7 cells treated with proteasome inhibitor MG132 (10μM) for 8h as indicated. * in western blotting was placed to indicate the position of USP22. **B** Ectopic expression of USP22 decreased the ubiquitination of ZEB1. Immunoprecipitation of ubiquitinated proteins from HCCLM3 cell extracts upon overexpression of USP22. Protein was harvested after MG132 (5μM) treatment for 3 h and ubiquitinated ZEB1 species were detected by western blotting with anti-His.

**Supplementary Fig. S6 Knockdown USP22 on the proliferation and migration of HCC cells. A, B** Western blotting assay demonstrated lentivirus-induced knockdown efficiency of USP22 in Huh7 and PLC/PRF/5 cells. * in western blotting was placed to indicate the position of USP22. **C** Knockdown of USP22 inhibited the growth of PLC/PRF/5 cells evaluated by Colony formation assay. **D** Effect of USP22 on cell migration evaluated by Transwell assay, scale bars, 250μm. *P* < 0.05 was considered statistically signiﬁcant.

**Supplementary Fig. S7 Knockdown of USP22 decreased *VEGFA* mRNA level in Xenograft tumors, but had no significant effect on *ZEB1* mRNA. A** Total RNA of xenograft tumors were extracted and the mRNA levels of USP22, VEGFA and ZEB1 were detected by qPCR. *P* < 0.05 is considered statistically signiﬁcant, ns indicates no statistical significance. **B** Periodic acid-Schiff (PAS) staining was used to identify the expression of mucopolysaccharide in each group in **Fig 6A.** **C** Correlation between USP22 and VEGFA protein expression in xenografts in **Fig. 6E**. Pearson r indicates the degree of correlation. **D** USP22 was positively correlated with ZEB1 in 24 HCC samples. Statistical plot of the correlation between ZEB1 and USP22 in HCC in **Fig. 7B**. Pearson r indicates the degree of correlation. *P* < 0.05 is considered statistically signiﬁcant.

**Supplementary Fig. S8 Analysis of the relationship between the overall survival of HCC patients and the expression of USSP22, ZEB1 or VEGFA. A** High expression of *VEGFA* positively correlates with poor clinical outcome in patients with HCC in 5 years. **B** HCC patients with both high expression of *VEGFA* and *USP22* have worse overall survival in 5 years. **C** High expression of *ZEB1* positively correlates with poor clinical outcome in patients with HCC in 5 years. **D** HCC patients with both high expression of *ZEB1* and *USP22* have worse overall survival in 5 years. All above data were downloaded from The Human Protein Atlas database. *P* < 0.05 was considered statistically signiﬁcant. HR>1 means that the indicated gene is considered to be a high-risk factor for HCC.
