# Supplementary material for: USP22 upregulates ZEB1-mediated VEGFA transcription in hepatocellular carcinoma
Source: Cell Death Dis. 2023 Mar 11;14(3):194. doi: 10.1038/s41419-023-05699-y (PMC10008583; doi:10.1038/s41419-023-05699-y)
Supplement: Supplementary file 5 — Supplementary Table [file 41419_2023_5699_MOESM5_ESM.docx]

**Table S1.** siRNA sequences against USP22

| Name | Sense (5’-3’) | Anti-sense (5’-3’) |
| --- | --- | --- |
| siUSP22 | CAAAGCAGCUCACUAUGAAdTdT | UUCAUAGUGAGCUGCUUUGdTdT |
| siZEB1#1 | GCUACUGGAGAUGGCAAUUTT | AAUUGCCAUCUCCAGUAGCTT |
| siZEB1#2 | GGAUCAACCACCAAUGGUUTT | AACCAUUGGUGGUUGAUCCTT |
| siZEB1#3 | GCAUACACCUACUCAACUATT | UAGUUGAGUAGGUGUAUGCTT |

**Table S2.** Real-time PCR primers for genes as indicated

| Name | Sense (5’-3’) | Anti-sense (5’-3’) |
| --- | --- | --- |
| *USP22* | CCATTGATCTGATGTACGGAGG | TCCTTGGCGATTATTTCCATGTC |
| *VEGFA* | CGAAACCATGAACTTTCTGC | CCTCAGTGGGCACACACTCC |
| *ZEB1* | TTACACCTTTGCATACAGAACCC | TTTACGATTACACCCAGACTGC |
| *MSRB3* | CGGTTCAGGTTGGCCTTCATT | GTGCATCCCATAGGAAAAGTCA |
| *VIM* | AGTCCACTGAGTACCGGAGAC | CATTTCACGCATCTGGCGTTC |
| *MMP14* | CGAGGTGCCCTATGCCTAC | CTCGGCAGAGTCAAAGTGG |
| *18S* | TTGACGGAAGGGCACCACCAG | GCACCACCACCCACGGAATCG |

**Table S3.** Primer sequences of VEGFA-promoter for Real-time PCR as indicated

| Name | Sense (5’-3’) | Anti-sense (5’-3’) |
| --- | --- | --- |
| Promoter I | TGGCCTGGTGGGAGC | AACCCCAGATGTTGCCAG |
| Promoter II | TTCCCAAAGGACCCCAGTCA | TGTGTCCCTCTGACAATGTGC |
| Promoter III | CCAGGGGTCACTCCAGGATTC | GAGAGCCGTTCCCTCTTTGC |
| Promoter IV | CGAGCCGCGTGTGGAAG | CCCGCTACCAGCCGACTTTT |
| Promoter V | CTGGTAGCGGGGAGGATCG | TCAAATTCCAGCACCGAGCG |
