# Supplementary material for: USP22 upregulates ZEB1-mediated VEGFA transcription in hepatocellular carcinoma
Source: Cell Death Dis. 2023 Mar 11;14(3):194. doi: 10.1038/s41419-023-05699-y (PMC10008583; doi:10.1038/s41419-023-05699-y)

Original western blots merged with molecular weight markers

Fig 1C

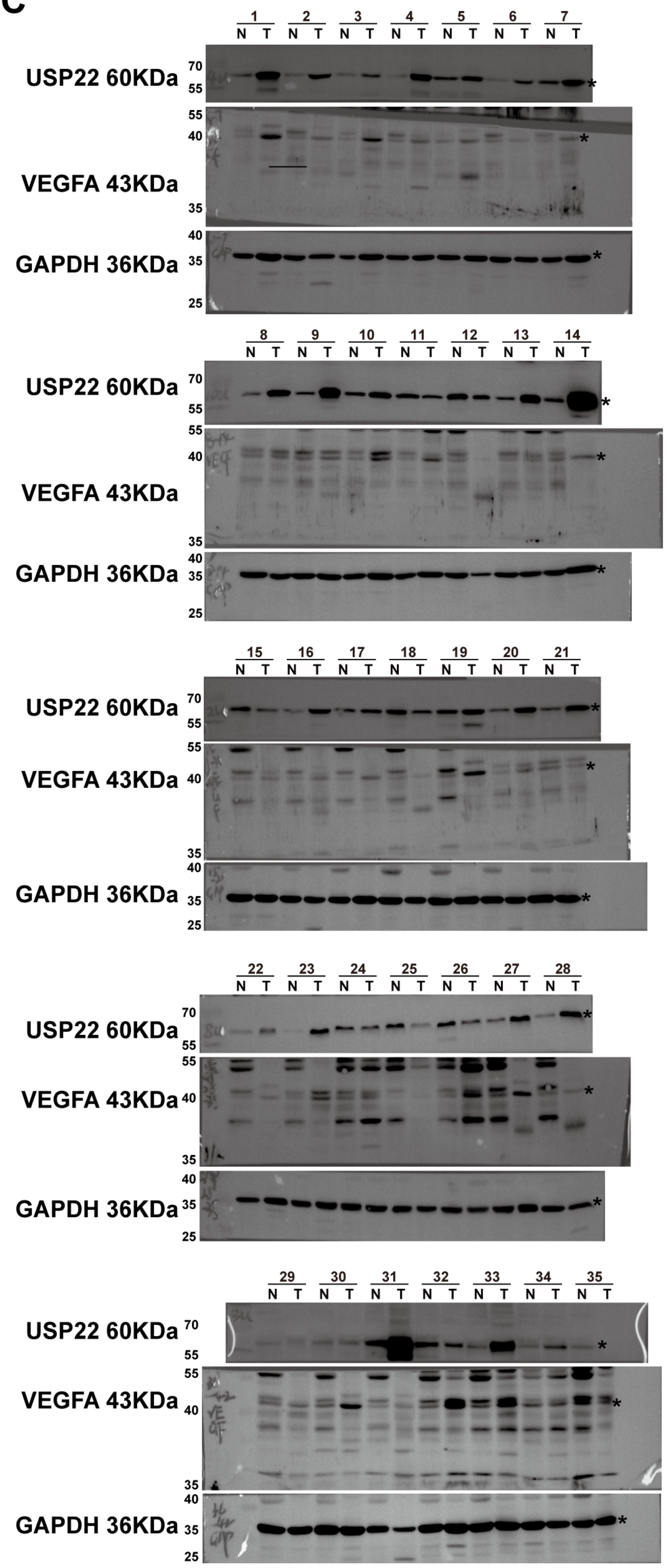

Fig 2A

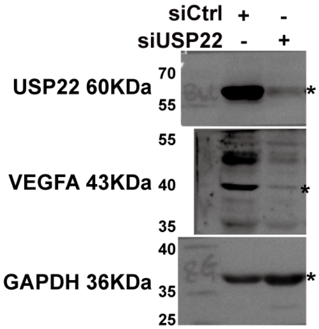

Fig 2B

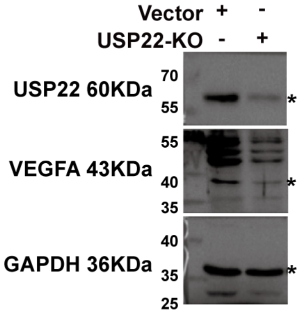

Fig 2C

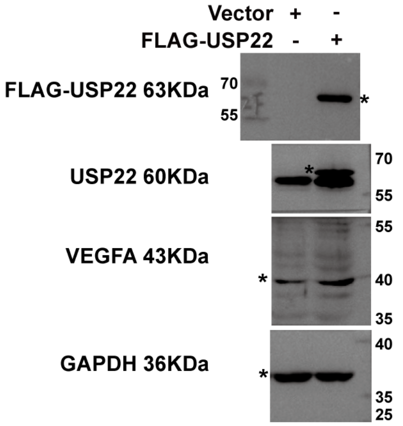

Fig 2I

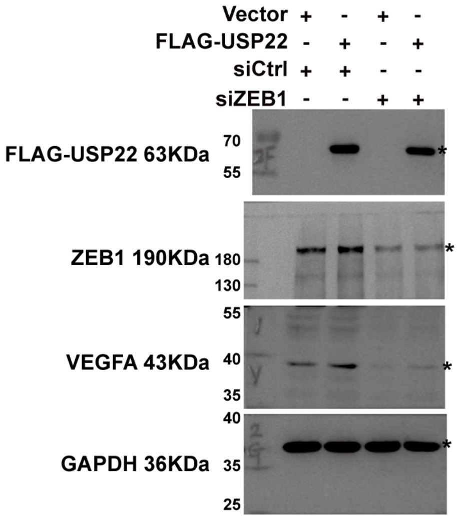

Fig 3A

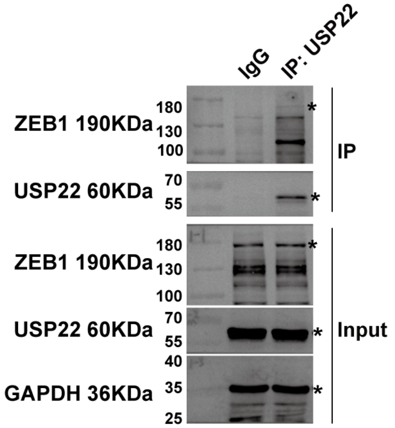

Fig 3C

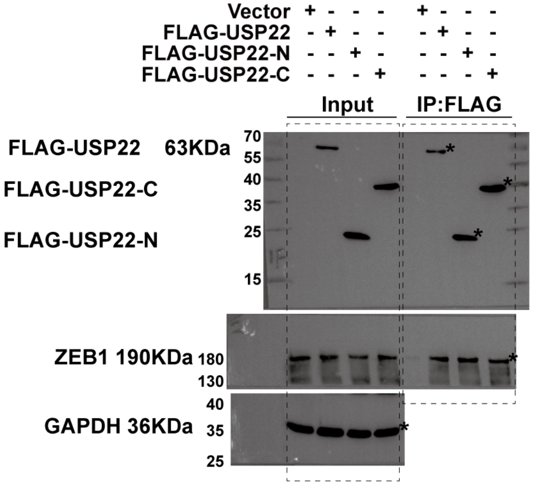

Fig 3D

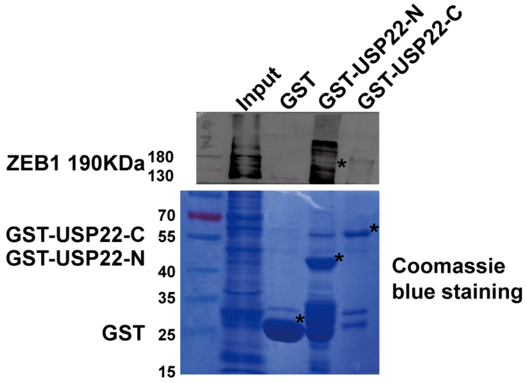

Fig 4A

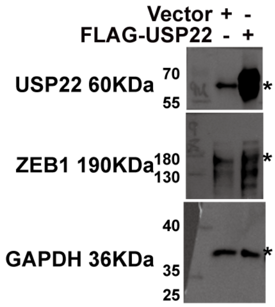

Fig 4B

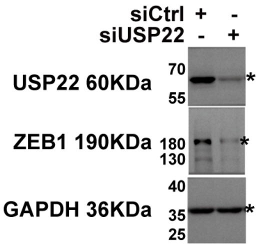

Fig 4D

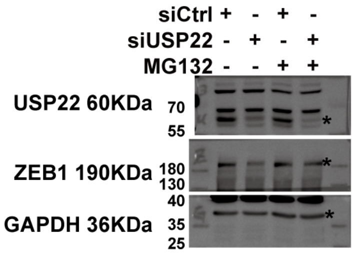

Fig 4E

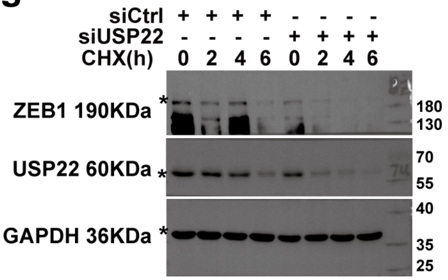

Fig 4G

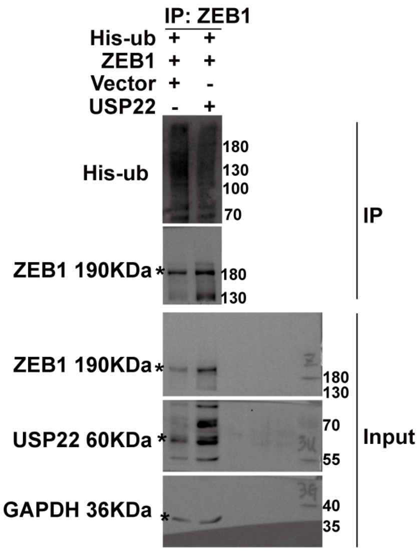

Fig 4H

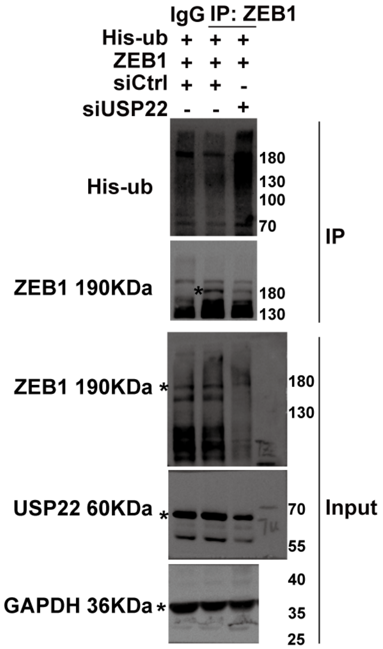

Fig 4I

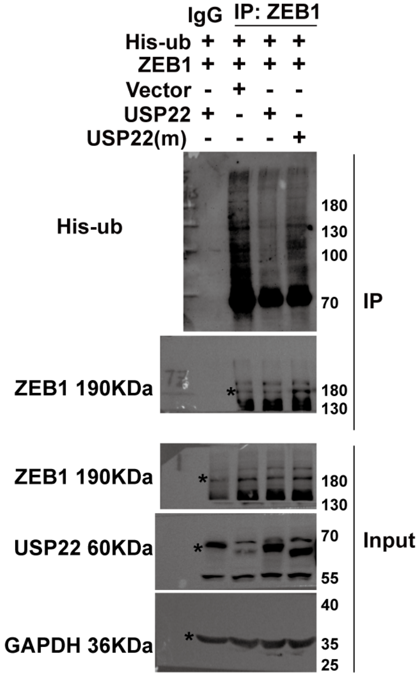

Fig 4J

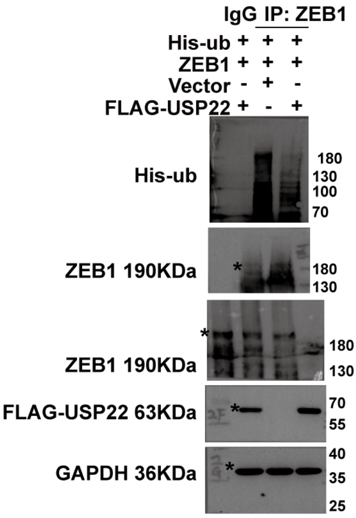

Fig 4K

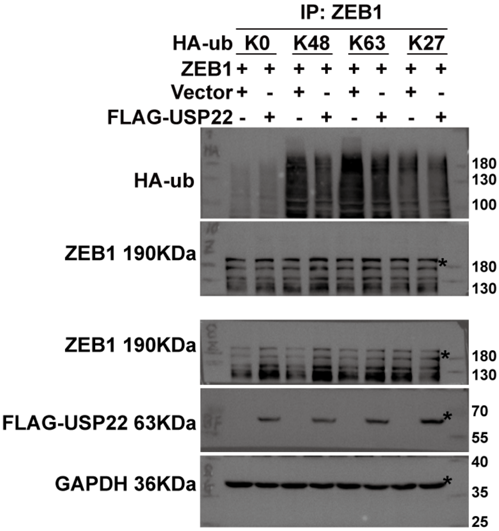

Fig 4L

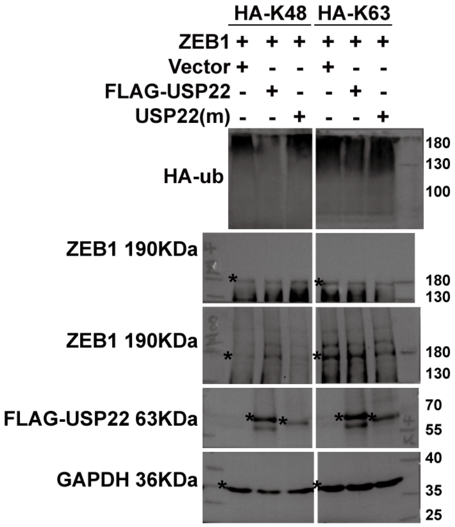

Fig 6E

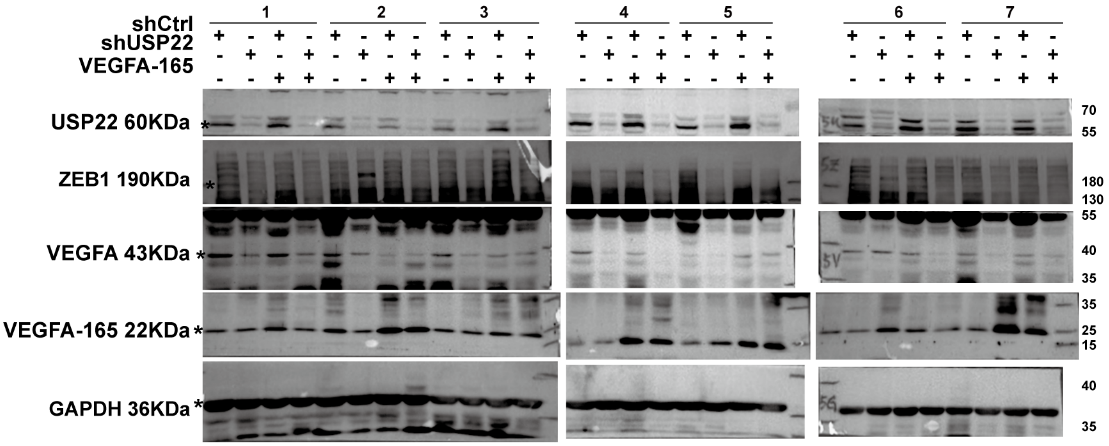

Fig 6J

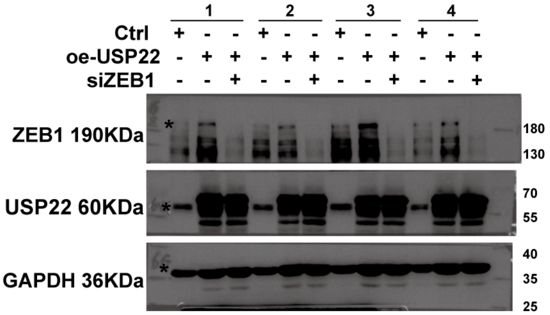

Fig S2B

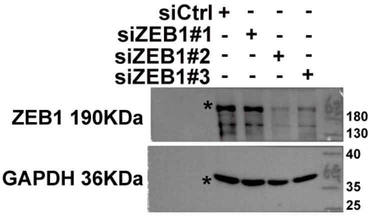

Fig S3A

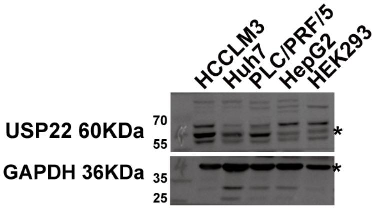

Fig S3B

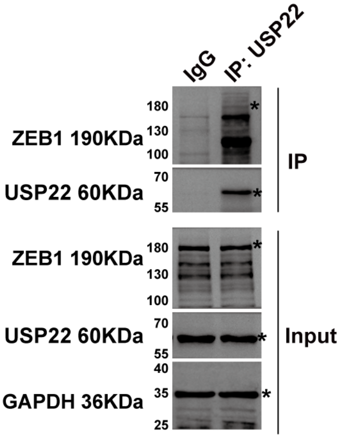

Fig S3C

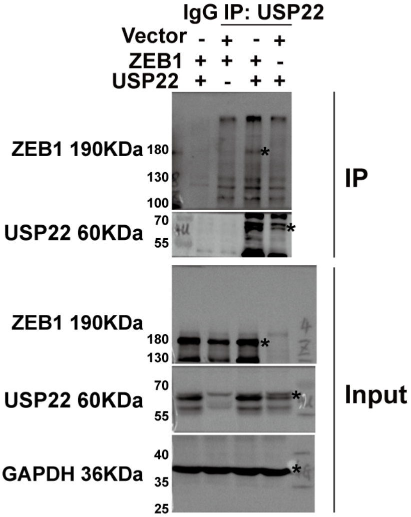

Fig S4C

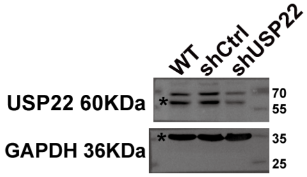

Fig S4D

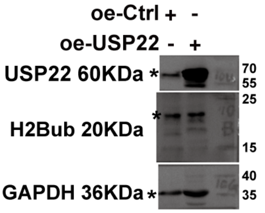

Fig S5A

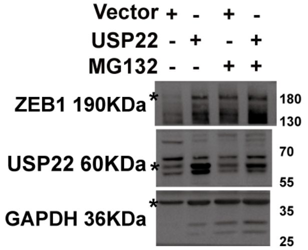

Fig S5B

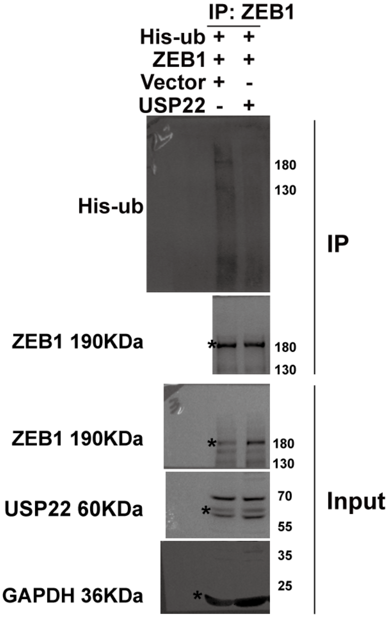

Fig S6A

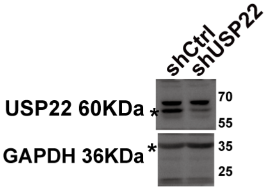

Fig S6B

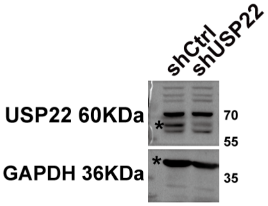

Supplement: Supplementary file 6 — Original Western blots [file 41419_2023_5699_MOESM6_ESM.pdf]
